# Enzyme-Free Phosphorylation with Kinetic Gating in a De Novo Coiled-Coil System

**DOI:** 10.1021/jacs.6c02162

**Published:** 2026-05-19

**Authors:** Simone M. Poprawa, Niklas L. Hoja, Clara Hipp, Reece O. King, Héctor Soria-Carrera, Derek N. Woolfson, Michael Sattler, Ville R. I. Kaila, Job Boekhoven

**Affiliations:** † Department of Bioscience, School of Natural Sciences, 9184 Technical University of Munich, Lichtenbergstrasse 4, Garching 85748, Germany; ‡ Department of Biochemistry and Biophysics, 7675 Stockholm University, Svante Arrhenius väg 16C, Stockholm 10691, Sweden; § School of Chemistry, University of Bristol, Bristol BS8 ITS, U.K.; ∥ Max Planck-Bristol Centre for Minimal Biology, Bristol BS8 ITS, U.K.; ⊥ School of Biochemistry, University of Bristol, Bristol BS8 ITD, U.K.; # Center for Protein Design, University of Copenhagen, Copenhagen DK-2100, Denmark

## Abstract

Phosphorylation is among the most ubiquitous and essential posttranslational modifications in biological systems. It is regulated by highly complex enzymatic networks. Here, we explore enzyme-free phosphorylation in a designed peptide system. Specifically, we use phosphorylation to modulate coiled-coil (CC) assembly and dynamics. This exploits a nonbiological reaction cycle to phosphorylate and dephosphorylate a histidine residue in an α-helix, enabling or disabling CC formation with a second helix. Dephosphorylation is kinetically gated—it is 25× faster in the CC compared to the nonassembled state. As a result, the cycle of phosphorylation, CC formation, dephosphorylation, and CC disassembly is ratcheted. The minimal synthetic phosphorylation cycle provides design principles and fulfills requirements to perform work mimicking the function of molecular motors, walkers, and pumps.

## Graphical abstract

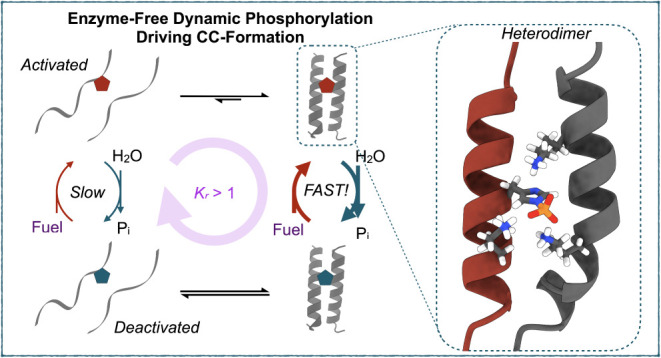

## Introduction

Life regulates the function of molecules and their assemblies away from equilibrium by transducing chemical energy. A central principle underlying this process is through reversible phosphorylation, which transiently creates and removes negative charges from proteins to alter their protein conformations and self-assembly, and the binding affinities of protein complexes.^1,2^ In cells, the hydrolysis of adenosine triphosphate (ATP) powers these of phosphorylation and dephosphorylation cycles, biasing molecular state populations and thereby controlling work and information flow. Classic examples include P-type ATPases whose catalytic aspartyl phosphorylation gates ion transport across membranes,^3^ kinase cascades where phospho-serine affects protein–protein interaction networks,^4^ and cell-cycle switches by writing multisite phosphorylation patterns.^5^ In cases where biology uses phosphorylation, it does not simply toggle between two protein conformations. Instead, it frequently uses kinetic gating to perform work, meaning that the kinetics of phosphorylation and dephosphorylation change only after the protein has undergone a specific mechanical or conformational transition.^6^ For example, in the Ca^2+^-ATPase, dephosphorylation is faster after the ATPase undergoes the large conformational change that brings calcium from one side of the membrane to the other.^3,7^ Such kinetic gating increases the overall efficiency of the pump and is found in many dynamic phosphorylation-dependent systems, such as pumps,^3,7–9^ motor proteins,^10–12^ and cytoskeletal polymers.^13–15^ Here, we set out to design a straightforward system to do this using α-helical coiled coils (Scheme 1A).

**Scheme 1:**
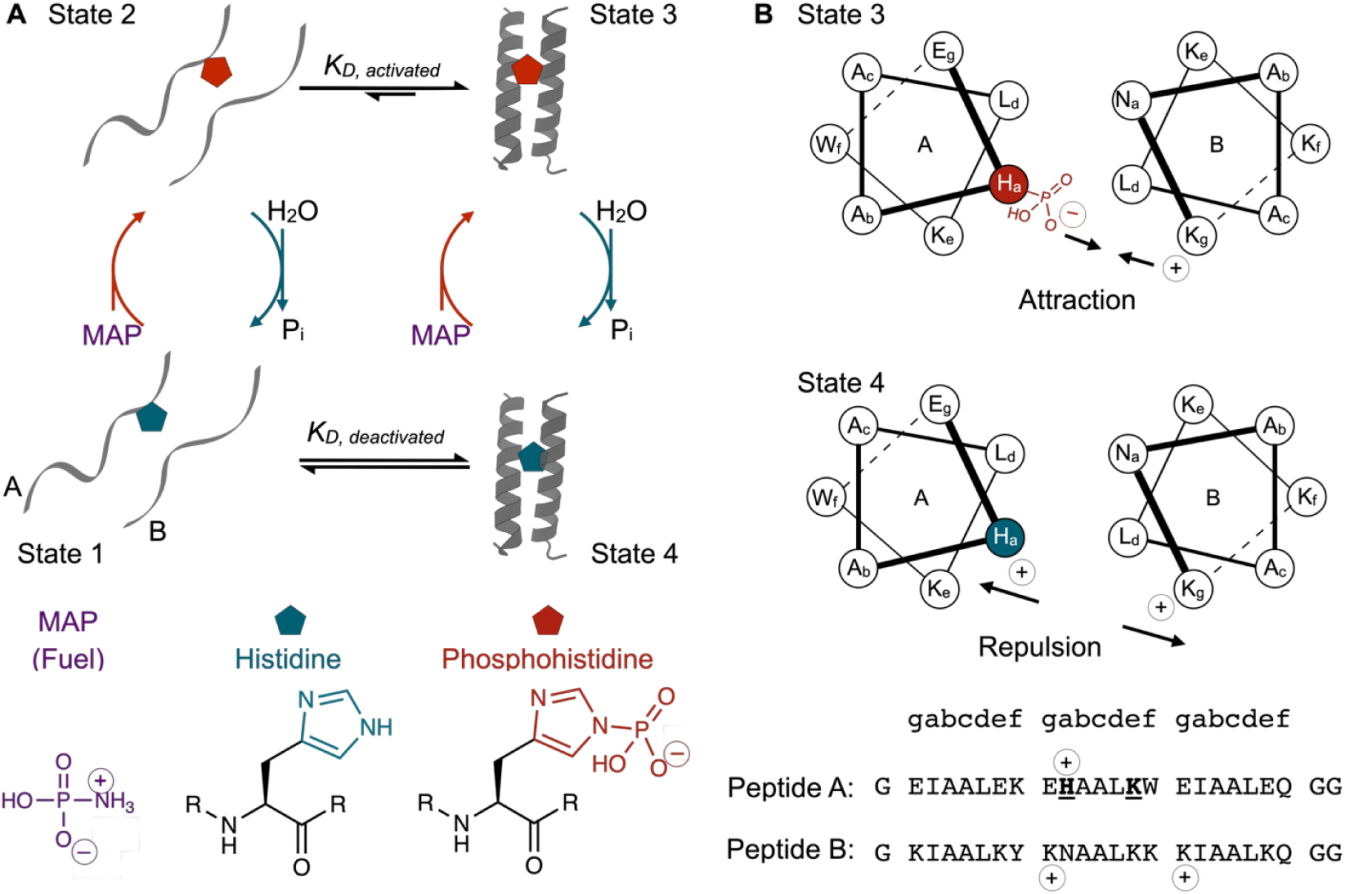
Design of the phosphorylation-controlled coiled-coil system; A) Reaction Cycle in which Mono-Amidophosphate (Map) Transiently Phosphorylates A Histidine Residue in Peptide A to Yield Activated Peptide A, While Dephosphorylation Restores the Unmodified State. The System can Access Four States: Free Deactivated Peptide (State 1), Free Activated Peptide (State 2), Activated Coiled-Coil (State 3), and Deactivated Coiled-Coil (State 4). Binding Equilibria Couple The Free and Bound States, While Activation and Deactivation Rates Depend on the Supramolecular Context. The Coiled-Coils in State 3 and 4 were Predicted by Alphafold3 ^76^ and Visualized with Protein Imager;^77^ B) Helical Wheel Representation of the Designed Heterodimer. Peptide A Carries A Central Histidine (h) At the A-position of the Second Heptad. In its Deactivated Form, This Cationic Residue Repels Lysine Residues on Peptide B (Repulsion, State 3). Upon Phosphorylation, The Negative Charge of Phosphohistidine Forms Stabilizing Electrostatic Interactions with the Lysines on Peptide B (Attraction, State 4), Favoring Coiled-Coil Assembly. Sequences of Peptides A and B are Shown Below, With Residues at A–G Positions Indicated

Among the protein interactions that can be regulated by phosphorylation, coiled coils offer particularly versatile examples and targets for design because they are programmable.^16–20^ Coiled-coil regions of proteins have heptad-repeat sequences, (*abcdefg*)_*n*_. These direct the formation of α-helices, which oligomerize in various ways based on sequence-to-structure relationships as shown in Scheme 1A.^21^ The *a* and *d* positions are usually hydrophobic. This encodes amphipathic helices that assemble via their *a/d* faces. The resulting interfaces are flanked by the *e* and *g* positions, which are often charged residues and contribute to coiled-coil specificity and stability through interhelical electrostatic interactions.^16,21–23^ Because coiled-coil binding free energies arise from many weak interactions, small, localized changes can shift their binding constants, inducing stimuli-responsive toggling between the assembled (bound) and disassembled (unbound) states. For example, the addition of salt^24–27^ or changing the state of protonation^26–28^ has been explored to modulate their interaction strength. Therefore, coiled coils provide powerful building blocks that convert site-specific chemical modifications into large changes in protein–protein interactions. However, in the examples above, the entire environment of the coiled coil is switched; for example, the pH or the salt content of the whole solution is changed. This approach contrasts with the biological dynamic phosphorylation approach, in which helices are locally affected, while retaining the same nonequilibrium environment. Thus, a dynamic coiled-coil that can be addressed chemically through nonequilibrium cycles would be a powerful tool to control protein functions, from switching scaffolds and condensates to force-producing assemblies.^29–33^

Recent work has demonstrated that dynamic phosphorylation can indeed switch coiled-coil interactions *in vitro* and in cell-free systems.^1,2,18,31,34–37^ In particular, elegant designs have shown enzyme-driven dynamic phosphorylation and dephosphorylation to toggle coiled-coil binding and downstream mesoscale behavior. These establish design rules to toggle coiled-coil formation via phosphorylation: for instance, placing phosphorylatable residues and sequences within the *g-a-d-e* interfaces can perturb the hydrophobic and electrostatic interactions to enable reversible, dynamic switching. Together, they make a compelling case that phosphorylation is a practical handle to program coiled-coil-mediated protein–protein interactions (PPIs). However, to date, all experimentally realized switching coiled-coil systems rely on enzymes (kinases/phosphatases) to activate and deactivate the coils.^1,18,20,21,34–36,38^ While effective, enzymatic control imposes constraints on peptide and protein design and their integration into systems chemistry. For example, enzymes, besides being bulky and having more complicated kinetics, require cofactors, such as Mg^2+^, in addition to ATP, and operate only under relatively narrow optimal temperature and pH.^39–42^ They often require a recognition motif around the phosphorylatable amino acid, which makes the protein design less straightforward.^31^ The bigger and more sophisticated the protein and the machine or motor are, the simpler and smaller the driving force should be, to cleanly and elegantly integrate into the system.^43,44^ Importantly, using kinases and phosphatases in this two-step system does not create a nonequilibrium steady state, which is required for an energy-harnessing system to eventually drive a molecular machine. These machines are driven by fuel-to-waste reactions to exploit the kinetic asymmetry/gating.^32,33^ From a systems-chemistry perspective, enzyme-free strategies provide a bottom-up framework for studying how chemical energy can regulate supramolecular states with minimal molecular complexity.

Indeed, recent, enzyme-free efforts in systems chemistry have emulated the nonequilibrium nature of biology using nonbiological fuels to regulate supramolecular systems.^43,45–48^ For example, carbodiimides,^49–51^ acyl phosphates,^52–55^ and amidophosphates^56,57^ have been used as fuels to regulate phase separation,^51,56,58–61^ and control supramolecular materials.^46,60,62–66^ In those designs, a molecule is activated by reacting with a chemical fuel, while this activated state spontaneously deactivates, typically through hydrolysis. Moreover, recent efforts in systems chemistry have emulated the kinetic gating^47,67,68^ to perform work with molecular machinery, such as pumps^32,33,69–71^ and motors.^32,47,71–73^ For example, kinetic gating can affect the fuel-driven activation or spontaneous deactivation after the molecular machine performs the next mechanical step. This concept leads to ratcheting of a cycle, which has been applied in molecular motors and pumps. However, coiled coils have not yet been used as general building blocks in such enzyme-free systems, despite their biological ubiquity.

In this work, we exploit an enzyme-free approach to regulate coiled-coil formation. We repurpose a previously introduced chemical reaction cycle^56^ that dynamically phosphorylates peptides and operates without enzymes to regulate AB-type heterodimeric coiled-coil formation.^74^ The cycle consumes monoamidophosphate (MAP) as a phosphorylating agent to install phosphate transiently on a histidine residue of peptide A, while dephosphorylation proceeds spontaneously through hydrolysis. Phosphorylation of the site is designed to favor binding of peptide A to a cationic peptide B, which stabilizes the heterodimer by ∼10 °C. As dephosphorylation occurs spontaneously, a nonequilibrium steady state is created in which strand A is dynamically toggled around four states (activated or deactivated, and in a coiled coil with B or not). We analyze the reaction kinetics to reveal a surprising degree of kinetic gating. Specifically, activation and deactivation are faster when peptide A is bound to peptide B. Moreover, molecular dynamics (MD) simulations and density functional theory (DFT) calculations help propose the underlying mechanistic principles revealing unique p*K*_a_ shifts upon the coiled-coil formation that drives the process. Taken together, we envision that our designs will provide powerful principles for protein design.

## Results and Discussion

### Design of the Enzyme-Free Dynamic Coiled Coils

We designed the enzyme-free dynamic coiled coils using the following considerations (Scheme 1A). First, at room temperature, coiled-coil formation should be unfavorable. Second, a judiciously placed histidine residue should disrupt coiled-coil formation through electrostatic repulsion. Third, histidine phosphorylation, inverting its charge from cationic to anionic, should favor coiled-coil formation. Fourth and finally, to avoid increased complexity of multiple phosphorylation, we opted for a heterodimer design in which only one peptide carries a single histidine. Based on these requirements, we designed a three-heptad, AB-type, heterodimeric coiled coil with a mostly anionic, phosphorylatable peptide A and a complementary, basic, and nonphosphorylatable peptide B (Scheme 1B). The initial designs were based on the *de novo* peptide system, CC-Di-AB, and we opted for only three heptads as they tend to have weaker interactions.^74,75^ We co-opted the phosphorylatable peptide A to be anionic using glutamic acids (E) on the *e* and *g* locations of all three heptads. We used isoleucine (I) and leucine (L) in the hydrophobic domain, specifically at the *a* and *d* positions of the heptad. Finally, on the second (middle) heptad, we mutated the isoleucine at location *a* to the phosphorylatable histidine (H). That way, the cationic histidine is in the middle of the triheptad and right in the hydrophobic domain, thus yielding maximum disruption to coiled-coil formation. We designed the B peptide to be cationic by introducing lysines (K) at the *g* and *e* positions of all heptads, and isoleucine and leucine at the *a* and *d* positions. That way, the anionic A peptide can form coiled-coils with the cationic B peptide. But, given the cationic histidine on peptide A, we expect the coiled-coil formation to be disrupted. Upon phosphorylation, the polarity of the histidine flips to anionic, and coiled-coil formation is expected to be favorable.^74^

With these design considerations, the coiled-coil system has four potential states (Scheme 1A). The peptides can be separated and unfolded with peptide A deactivated (1), similarly, they can be disengaged but with peptide A activated (2), they can be assembled in a coiled-coil state with the peptide A activated (3), or similarly, they can be assembled but with the peptide A deactivated (4). The toggling from one state to the other can occur under thermodynamic (horizontal arrows in the scheme, supramolecular coiled-coil formation and disengagement) or kinetic (vertical arrows in the scheme, activation/phosphorylation and deactivation/dephosphorylation) control. The thermodynamic part is based on the interaction strength, defined by the coiled-coil dissociation constants *K*_*D*_, of the activated and deactivated states. The kinetic toggling is regulated by the phosphorylation and dephosphorylation of the histidine in the free and coiled-coil states.

### Thermodynamic Control over the Coiled-Coil Formation

We found that the first design of peptides A and B interacted too strongly at room temperature despite being only a three-heptad heterodimer (peptide A1 and B1, Supporting Table 1, Supporting Figure 1). Specifically, circular dichroism (CD) spectroscopy revealed a melting temperature *T*_*m*_ of 64.0 ± 1.0 °C at 50 μM peptide A1 and B1 each in 15 mM 4-(2-hydroxyethyl)-1-piperazineethanesulfonic acid (HEPES) buffer at pH 6.5 (Supporting Figures 3A and C). As this would violate our first requirement (coiled-coil formation should be unfavorable in the deactivated state), we mutated the glutamic acid in the second heptad of peptide A to a lysine (Scheme 1B, Supporting Table 1, Supporting Figure 1). This mutation increases the overall charge of peptide A to −2, thus expected to weaken the interaction with peptide B. We found that these peptides still interacted strongly with a *T*_*m*_ of 44.6 ± 0.3 °C (Peptide A and B1, Supporting Figures 3A and C). Therefore, we also mutated peptide B1, replacing the middle isoleucine with asparagine (N), which is known to further weaken interactions and is compatible with dimeric coiled-coils.^21^ This yielded our final set of peptides A and B (Scheme 1B, Supporting Table 1, Supporting Figures 1 and 3A and C), with a *T*_*m*_ close to room temperature (*vide infra*). The reaction conditions of our system were 15 mM HEPES at pH 6.5, without the addition of salt, except for adjusting the pH, which allowed us to monitor a change with CD spectra induced by activating peptide A (Supporting Figure 5). We chose to work at pH 6.5 based on an optimization assay from our previous work.^56^

Initially, we investigated the thermodynamic contribution of the four-state system (Figure 1A). We recorded CD spectra of 50 μM peptide A and B alone (Figure 1B, dotted lines) and combined as the coiled-coil system (Figure 1B, straight lines). Neither peptide A nor B alone showed the characteristic negative peaks at 208 and 222 nm, indicative of α-helices. Indeed, the calculated helicity from the CD spectra^78^ for peptide A was 5.7% and for peptide B 4.0% (Supporting Figure 6). In contrast, the combination of both peptides resulted in the characteristic peaks, increasing helicity to 36.1%, indicating an α-helical structure consistent with coiled-coil formation (Supporting Figure 6). This was supported by more dispersion in the ^1^H–^15^N-heteronuclear single quantum coherence (HSQC) nuclear magnetic resonance (NMR) spectra at low temperature (Supporting Figure 7). These data point toward some interaction between the two peptides, leading to partial α-helix and coiled-coil formation. With the two peptides combined, we recorded melting curves at the characteristic 222 nm wavelength from 5 to 90 °C. The deactivated system has a melting temperature of 26.6 ± 0.4 °C (Figure 1C, blue curve). Put differently, at room temperature, 58% of our coils are engaged, and 42% are free (Supporting Figure 3B).

**Figure 1:**
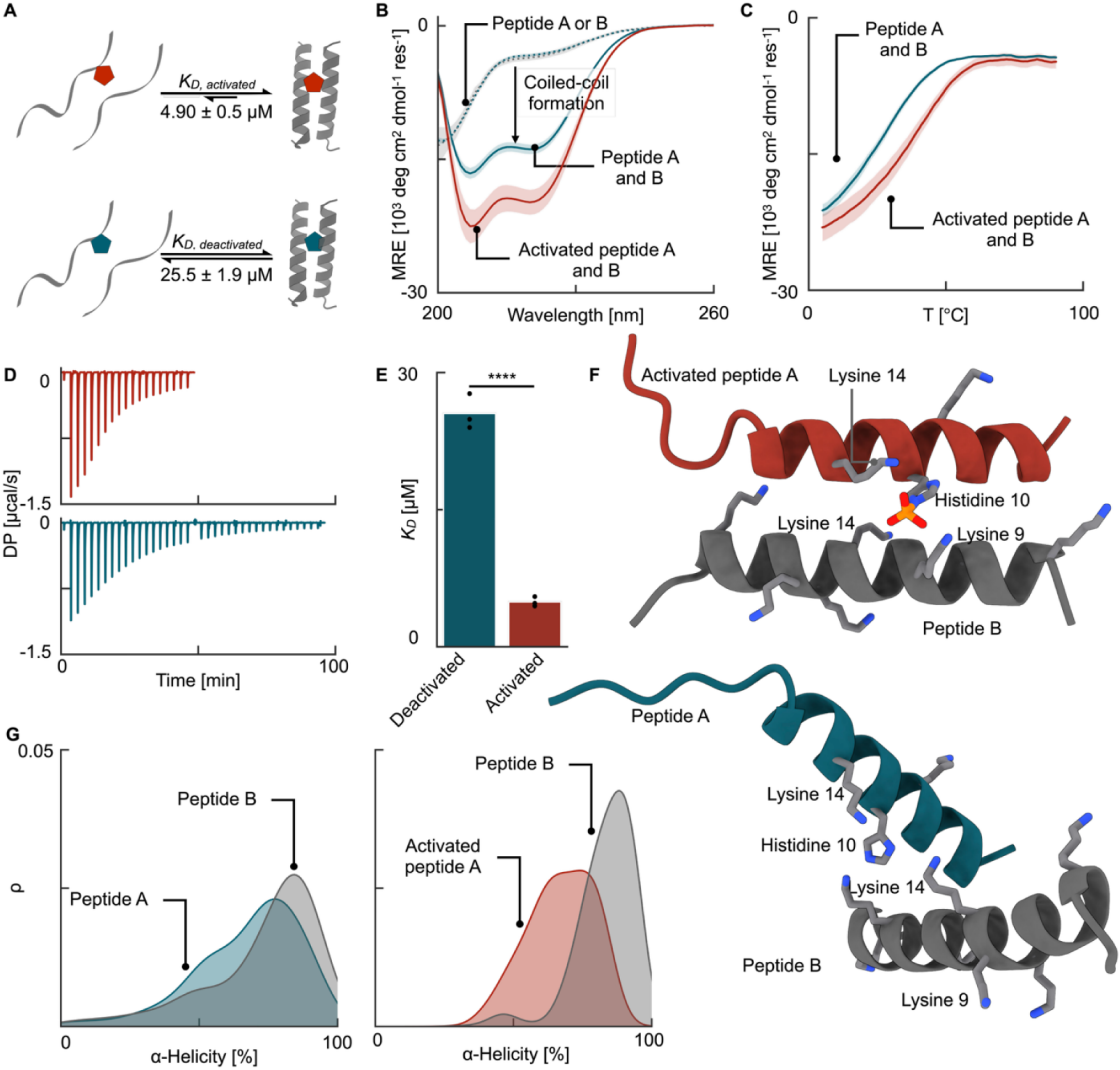
Thermodynamics of the phosphorylation-enhanced coiled-coil formation.A) Schematic representation of the four-state system: peptide A exists in a deactivated (blue hexagon) or activated (red hexagon) state, and each can be either free or engaged in a coiled-coil with peptide B. B) Circular dichroism (CD) spectra of peptide A (dotted black), peptide B (dotted gray), the A–B mixture (straight blue), and the activated A–B mixture (straight red) at 50 μM in 15 mM HEPES buffer at pH 6.5. (*n* = 3) Neither peptide alone shows significant helicity, whereas the combination exhibits the characteristic minima at 208 and 222 nm, indicative of coiled-coil formation, and the combination of the activated peptide A and B showed an even more pronounced coiled-coil formation and increase in helicity. (*n* = 3) C) Thermal denaturation curves at 222 nm reveal melting temperatures (*T_m_*) of 26.6 ± 0.4 °C for the deactivated coiled-coil (blue) and 36.4 ± 0.5 °C for the phosphorylated, activated coiled-coil (red), indicating increased stability upon phosphorylation. (*n* = 3) D,E) Isothermal titration calorimetry (ITC) of peptide B titrated into peptide A yields dissociation constants of *K*_*D*,deactivated_ = 25.5 ± 1.9 μM and *K*_*D*,activated_ = 4.90 ± 0.5 μM, confirming an increase in affinity upon phosphorylation. (D: *n* = 1, E: *n* = 3) F) Molecular dynamics simulations indicate that the phosphate group on histidine forms stabilizing electrostatic interactions with Lys14 on peptide A and Lys9 and Lys16 on peptide B, thereby reinforcing the coiled-coil formation (top). Whereas the nonactivated coiled-coil system for a loosely associated state and do not form a stable coiled-coil system (bottom). G) Density distributions of α-helical content for peptide A (blue), activated peptide A (red), and peptide B (gray). The activated dimer overall stabilizes the coiled-coil formation.

Next, we compared the thermodynamic behavior of peptide A to that of the activated peptide A. To achieve this, we prepared the phosphorylated peptide A by incubating it with an excess of MAP for 2 days, and separated the phosphorylated and the nonphosphorylated species by preparative high-performance liquid chromatography (HPLC) (Supporting Figure 2). Again, activated peptide A alone did not show any α-helical character (Supporting Figure 4). However, when mixed with peptide B, the calculated helicity of the combined coiled-coil system of activated peptide A and peptide B, as measured by CD spectroscopy, increased to 58.9% (Supporting Figure 6), and the *T*_*m*_ shifted to 36.4 ± 0.5 °C (Figure 1C), indicating a significantly stronger interaction between the activated than the deactivated state. The phosphorylated peptide A is likely dephosphorylated during the recording of the melting curve, leading to peptide A with reduced binding affinity. Therefore, we assume the *T*_*m*_ of the activated peptide is likely an underestimate. To quantify the interaction strength between the peptides, we performed isothermal titration calorimetry (ITC) experiments on the activated and deactivated peptide A with peptide B (Figure 1D). We found peptide A and peptide B interact with a dissociation constant for the deactivated system (*K*_*D*,*deactivated*_) of 25.5 ± 1.9 μM and a stoichiometry of 0.89 ± 0.1, further corroborating the coiled-coil formation (Figure 1E, Supporting Figure 8). In contrast, the *K*_*D*_ for the activated state (*K*_*D*,*activated*_) decreased to 4.90 ± 0.5 μM with a stoichiometry of 0.59 ± 0.1 (Figure 1E, Supporting Figure 9). We conducted ITC experiments to determine the dissociation constants and binding stoichiometries of the deactivated peptide A and B coiled-coil pair, along with an approximation of the dissociation constant of the activated peptide A and B coiled-coil pair for direct comparison. We acknowledge that ITC is usually used to investigate the binding energies of static, nonreacting molecules. In our case, the activated peptide A undergoes hydrolysis. The ITC measurement therefore reports an average over two coexisting populations with distinct binding affinities for peptide B, which change over time and with the injected amount of peptide B. Additionally, the enthalpy of hydrolysis of the activated peptide to peptide A and phosphate contributes a background thermal signal, “contaminating” the measured parameters. Therefore, the *K*_*D*_ must be a lower estimate, an effective population-averaged quantity, of the actual K_D_. The increased binding (lowering of *K*_*D*_ value) with activated peptide A supports our hypothesis that phosphorylation increases peptide–peptide interactions by strengthening the binding.

To provide a more detailed, mechanistic understanding of the coiled-coil formation of peptides A and B and their activated counterpart, we performed atomistic molecular dynamics (MD) simulations. Specifically, peptides A and B were embedded in a TIP3P water box in α-helical conformations approximately 20 Å apart and simulated for 500 ns in four replicas of each conditions: deactivated, activated, parallel, and antiparallel (Supporting Table 2, Supporting Figure 14 and 17–28, Supporting Movies 1–4). In line with our data described above, the MD simulations indicated that peptide A and peptide B did not form any stable coiled-coil-like interactions. Instead, the peptides remained largely separated in monomeric or loosely associated states during the simulations (Figure 1F bottom, Supporting Movies 1 and 2), while the initial orientation of the peptides, either parallel or antiparallel, had only a minor influence on the trajectories of the simulations.

In contrast, activated peptide A and peptide B rapidly formed a dynamic, yet stable coiled-coil-like structure over the entire MD simulations (Figure 1F top, Supporting Movies 3 and 4). The peptides engaged at distances and angles consistent with parallel, dimeric coiled coils (Supporting Figures 18–25). From the MD simulations, we calculated the distributions of peptides A and B in α-helical configurations. We find that both peptides A and B had some α-helical character when interacting with each other, while the presence of peptide B and activation of peptide A leads to a significant stabilization of the α-helical conformations of both peptides. This suggests that the α-helical structure of the coiled-coil state is stabilized by the phosphorylation (Figure 1G, Supporting Figures 16 and 18–28).

### Kinetics of the System

Next, we investigated the kinetics of our four-state model, namely the activation and deactivation processes. To understand the reaction kinetics of peptide A in its free, noncoiled-coil state, we first focused on its kinetics in the absence of peptide B as a model for the peptide A free in solution (Figure 2A). We added 12.5 mM potassium monoamidophosphate (MAP) to 50 μM of peptide A in 15 mM HEPES at pH 6.5 and monitored the formation of the activated peptide A using high-performance liquid chromatography (HPLC) (Supporting Figure 10). We found that it increased to approximately 2.3 μM within the first 40 h. Then, due to the dynamic phosphorylation and the loss of MAP as a fuel, it decreased over time (Figure 2A and C, Supporting Figure 11). By ^31^P NMR, we measured the consumption of the MAP and found that it decayed with a half-life of 13.0 h (Supporting Table 3). Based on the combined data sets, we determined the dephosphorylation rate constant of the activated peptide A in a regime where no fuel was present, i.e., where the only reaction acting on activated peptide A was its deactivation. We found it to be 0.011 h^–1^, which translates to a half-life of 63.0 h. To predict the activation rate constant of peptide A, we updated a previously developed kinetic model (see the section on the Kinetic Model and Supporting Tables 2 and 3 in the Supporting Information). The kinetic model describes the concentration of activated and deactivated peptide A, MAP, and waste. We used the hydrolysis rate constant of MAP, determined empirically by ^31^P NMR (*k*_0_, Supporting Table 2). We also used the empirically determined deactivation rate constant, explained above (*k*_–1_). With these rate constants, we fitted the curves to obtain an estimate of the activation rate constant at 0.25 M^–1^ h^–1^ (*k*_1_, Supporting Table 3, Supporting Figure 13).

**Figure 2:**
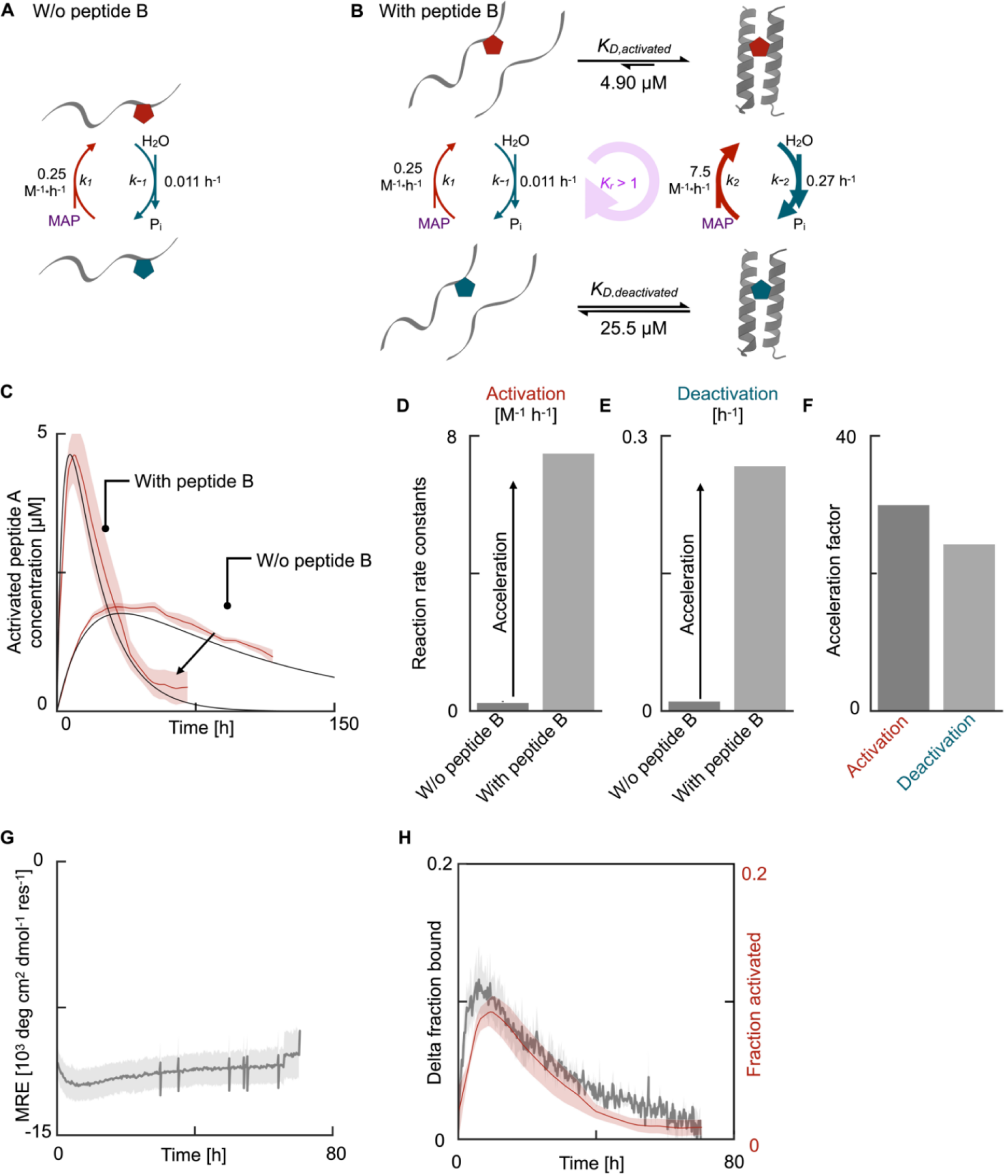
Kinetics of the phosphorylation-enhanced coiled-coil formation. A,-B) Schemes of the activation and deactivation kinetics with and without peptide B. C) Concentration of phosphorylated peptide A over time after addition of 12.5 mM MAP fuel to 50 μM peptide A with and without peptide B, monitored by HPLC. In the absence of peptide B, activated peptide accumulates transiently to ∼1.9 μM after 31 h before decaying as fuel is depleted. In the presence of peptide B, activated peptide accumulates transiently to ∼4.2 μM after 8.4 h before decaying as fuel is depleted. Kinetic model fit (black line) to the experimental data (red line). (*n* = 3) D) Activation and E) deactivation rate constants of peptide A with and without peptide B. F) Acceleration factor of the activation and deactivation of the bound state compared to the unbound state. G) The MRE over time recorded with CD spectroscopy at 222 nm shows a transient increase in helicity upon fueling, H) corresponding to a change in bound fraction from 0 to 11%, which relaxes as fuel is consumed (gray line). The close overlap of CD and HPLC (red line) kinetics indicates that nearly all phosphorylated peptide A is bound to peptide B. (*n* = 3).

To our surprise, when we performed the same kinetic experiment with 50 μM peptide B present, the maximum concentration was reached after approximately 6 h, while the overall cycle lasted much shorter at roughly 75 h (Figure 2B and C, Supporting Figure 12). Besides, the maximum concentration of activated peptide A was almost double that without peptide B at 4.5 μM activated peptide. Using analytical HPLC, we determined the deactivation rate constant of purified activated peptide A in the presence of peptide B to be 0.27 h^–1^, corresponding to a half-life of 2.6 h (Supporting Table 3). In other words, the observed deactivation rate increased 25-fold in the presence of peptide B (Figure 2E and F). Moreover, despite the increased deactivation, we observed a higher maximum concentration of activated peptide A, from which we conclude that the activation rate must also be accelerated by the presence of peptide B (Figure 2C). Such an increase in activation and deactivation depending on the mechanical state of the system implies some form of kinetic gating.

To determine the rate constants of activation and deactivation of peptide A in the presence of B, we adjusted the kinetic model introduced above and made the following assumptions (see the section on the Kinetic Model and Supporting Tables 2 and 3). First, we assumed that the activation rate constant of unbound peptide A was equal to the rate constants in the absence of peptide B, which we determined above. Second, we assumed all activated peptide A was associated with peptide B. This assumption is confirmed by circular dichroism spectroscopy data discussed later. With this latter assumption, we thus assume all deactivation occurs in the coiled-coil state, and we can thus state that the observed deactivation rate constant we measured above is the deactivation rate constant of the activated peptide A associated with peptide B (Figure 2B, *k*_–2_). As a third assumption, we stated that 42% of the peptide A was not bound, because the experiments were performed close to the melting temperature of the coiled coil formed by the deactivated peptide A (Supplementary Figure 3B). Thus, the activation of this fraction of the peptide was unaffected by the presence of peptide B. In contrast, the remaining 58% of the peptide that was bound must have a drastically increased activation rate constant, because the observed activation was much faster compared to that without peptide B. We fitted the observed kinetics with these assumptions and found the data could be fitted when we adjusted the activation rate constant to be 30 times faster than the bound peptide (Figure 2D and F), i.e., *k*_2_ = 7.5 M^–1^ h^–1^.

Using CD spectroscopy, we measured the response of peptides A and B to fuel at 222 nm (Figure 2G). We observed a transient decrease in the mean residual ellipticity (MRE) over the course of the 75 h reaction cycle, corresponding to an increase in coiled-coil formation. We normalized the MRE to the fraction with respect to the individual melting curves of each replicate (eq 1). From these data, we calculated a change in the bound fraction (delta fraction bound, eq 2) that rapidly peaked and then decayed again until it reached 0. From the HPLC data, we calculated the percentage of activated peptide and observed a fitting overlap between the two data sets (Figure 2H). Based on this overlap, we conclude that almost all peptide A that was activated was also bound to peptide B.

1 $$\mathrm{fraction}=\frac{{\mathrm{MRE}}_{t}-{\mathrm{MRE}}_{\mathrm{unbound}}}{{\mathrm{MRE}}_{\mathrm{bound}}-{\mathrm{MRE}}_{\mathrm{unbound}}}$$

2 $$\mathrm{delta fraction bound}=\left({\mathrm{fraction}}_{t_{0}}-{\mathrm{fraction}}_{t_{0+x}}\right)\times 100$$

From the combined data, we conclude that the system’s kinetics are biased by kinetic gating, which can act as a ratchet for the cycle’s directionality. In other words, from the four rate constants and two equilibrium constants, we can calculate whether a peptide cycles preferentially clockwise or counterclockwise through the four states, i.e., the system’s ratcheting constant (*K*_*r*_). With the definition of our system, if *K*_*r*_ is >1, it cycles clockwise; if it is <1, it runs counterclockwise. We determined it to be 4.3 using the adjusted equation (eq 3).^47^

3 $$K_{r}\approx q=\frac{k_{1}\times k_{-2}\times K_{D,deactivated}}{k_{2}\times k_{-1}\times K_{D,activated}}$$

This value corresponds to a slight clockwise direction of the system. The use of this equation requires the following considerations. Peptide B is not activated, but is part of the thermodynamics of the system and influences the kinetics of association of the coiled coils. We also assumed that all reactions do not occur reversibly at a significant rate, *i.e*., the fuel is “irreversibly” consumed (Supporting Information section on ratcheting constant).

### Mechanism of Kinetic Gating

From the data above, the mechanical state of peptides A and B (coiled-coil or not) strongly affects the kinetics of activation and deactivation. Both are drastically accelerated by coiled-coil formation with peptide B (Figure 3A). Next, we sought the mechanism behind the kinetic gating. The helical wheel representation of the coiled coils shows which nucleophilic or electrophilic amino acids are in proximity to the histidine, namely glutamic acid, lysine, and asparagine (Figure 3B). To investigate which residue could affect phosphorylation and dephosphorylation, we determined their activity in the absence of peptide B, but with a large excess of mimetics of the possible sites. Initially, we added the side-chain mimetics from the start of the cycle and initiated the reaction with MAP to assess their effects on peptide A activation and the overall cycle. In another experiment, we added the side-chain mimetics to the purified, activated peptide A to investigate their influence on hydrolysis. We used 1 M acetamide to mimic the asparagine side chain, 1 M acetic acid for glutamic acid, and 1 M ethylenediamine (expressed in amine functional group) to replicate lysine at pH 6.5. We used a 2.6 M ionic strength with all compounds to ensure comparable results and 2.6 M ionic strength as control. First, we compared the apparent yield of activated peptide A after an average of 7.8 h, which corresponds to the maximum yield of the system containing peptide A and B (Figure 3C). The concentration of activated peptide A in the absence of peptide B at this time was 0.75 μM. With 1 M of acetamide, no significant difference was observed. In contrast, with 1 M acetic acid and 1 M ethylene diamine, there is no visible formation of activated peptide A. These findings suggest that amines and/or carboxylic acids (i.e., lysines and/or glutamic acids) in proximity to the phosphorylated histidine could influence phosphorylation and dephosphorylation.^79,80^

**Figure 3:**
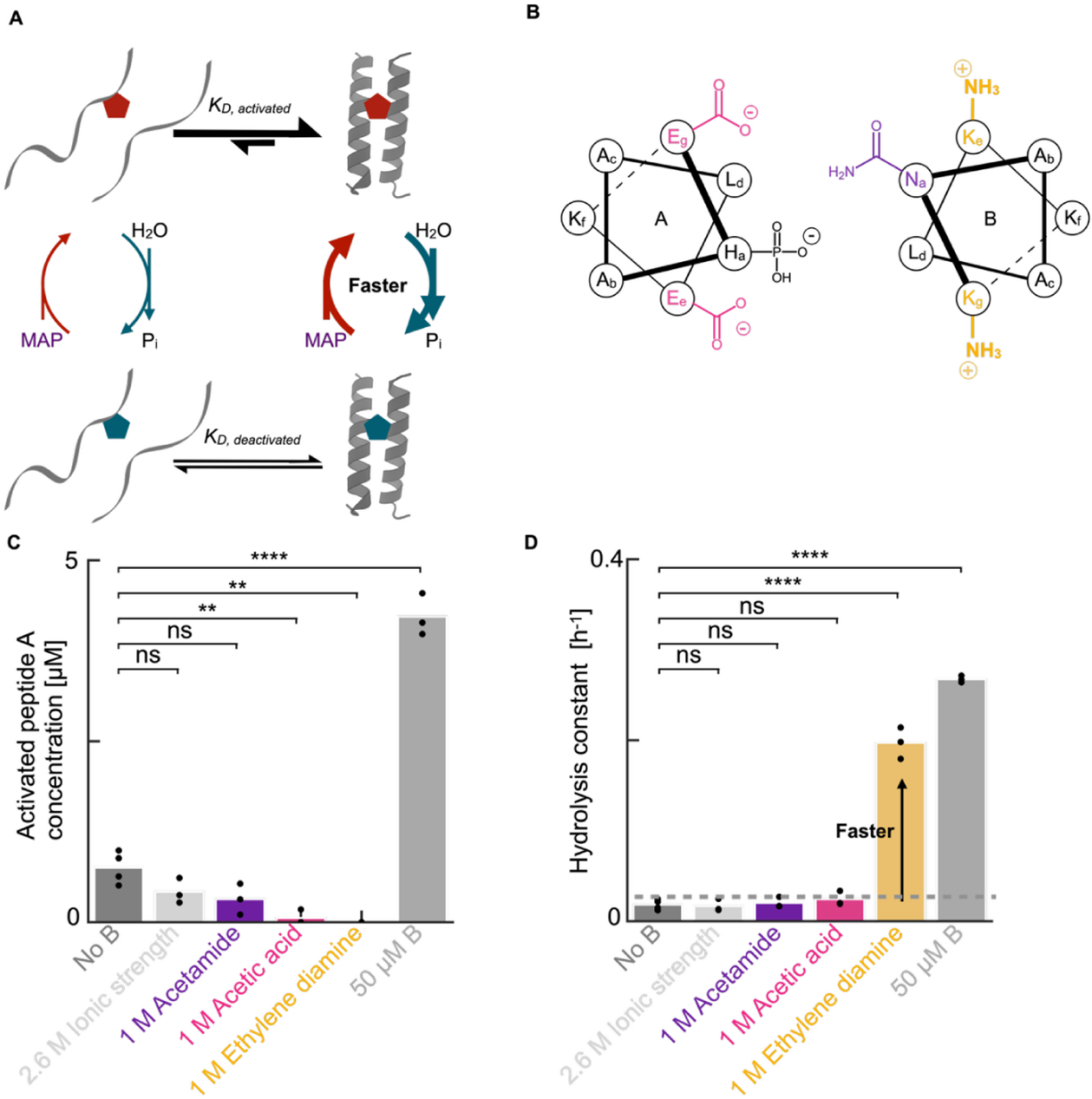
Catalytic activity of the coiled-coil on activation and deactivation. A) Schematic representation of the kinetics of phosphorylation and dephosphorylation in the presence of peptide B. B) Helical wheels of peptide A and B with potential catalytically active side chains. C) Concentration of activated peptide A 7 h after the start of the reaction in the presence of excess additives to mimic the high concentration environment of the side chains, when peptide B is present. No significant change for the addition of 2.6 M ionic strength, 1 M acetamide (mimic for asparagine, purple), acetic acid (mimic for glutamic acid, magenta) and ethylene diamine (mimic for lysine, yellow), but peptide B. (*n* = 3) D) Hydrolysis rate constant for activated peptide A in the presence of excess additives to mimic the high concentration environment of the side chains, when peptide B is present. No significant change for the addition of 2.6 M ionic strength, 1 M acetamide (mimic for asparagine), and acetic acid (mimic for glutamic acid), but ethylene diamine (mimic for lysine), and peptide B. (*n* = 3).

To determine whether these additives specifically affect phosphorylation or dephosphorylation, we screened the same additives and added them to purified phosphorylated peptide A. Using HPLC, we monitored the concentration of activated peptide A over time and determined the apparent hydrolysis rate constant (Figure 3D). We compared these rate constants to the deactivation rate constant we found above in the presence of peptide B. None of the additives increased the deactivation rate constant compared to no peptide B, except for the addition of diethylene diamine, which we used as a model for the local lysine. From these data sets, we suspect that local lysines on peptide B are responsible for the dramatic changes in activation and deactivation kinetics.

To gain molecular insights into the mechanism of kinetic gating, we conducted atomistic molecular dynamics (MD) simulations of the system (Scheme 1B, Figure 4A, see Methods). The MD simulations suggest that activated peptide A and peptide B form a coiled-coil structure in which the phosphorylated histidine interacts with lysine 16 and lysine 9 on peptide B and lysine 14 on its own coil (Figure 4A, B), and resulting in stable coiled-coil formation during the MD simulations. In contrast, the dephosphorylated state results in a higher population of the dissociation helices (Supporting Figure 26–27).

**Figure 4:**
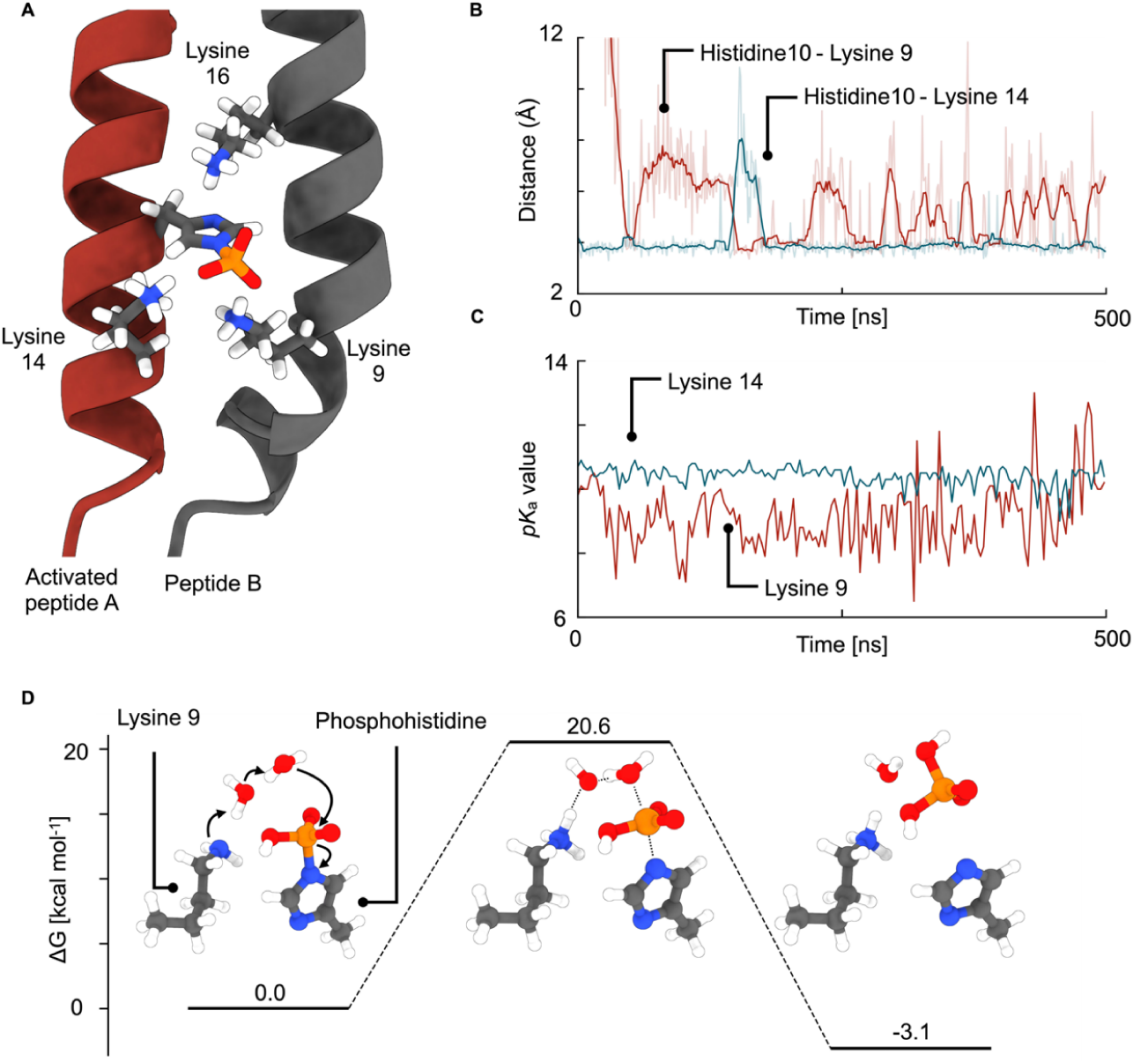
Putative mechanism of phosphate hydrolysis in the coiled-coil state. A) Structural MD snapshot of the coiled-coil interface, illustrating the interaction network between phosphohistidine 10, and lysine 14 on peptide A and lysine 9, and lysine 16 on peptide B. B**)** Dynamics and distribution of distances between the phosphorylated histidine on peptide A and the surrounding lysine 9 on peptide B, lysine 14 on peptide A, during 500 ns of MD simulation. The data show stable interactions between phosphohistidine 10 and the neighboring lysine residues. C) Calculated p*K*_a_ values of lysine 9 on peptide B and lysine 14 on peptide A during the MD trajectory, resulting in a pronounced p*K*_a_ decrease of lysine 9. D) DFT model and energetics of the phosphate hydrolysis reaction showing the optimized reactant, transition, and product states and corresponding free energies (ΔG, kcal·mol^–1^) (Supporting Table 5: Q6 for further details). The DFT calculations suggest that a lysine 9 on peptide B-mediated deprotonation of a water molecule, following an S_N_2-type nucleophilic attack of the hydroxide on the phosphate center, and resulting in the cleavage of the P–N bond and release of inorganic phosphate.

To probe if the proximity of the lysines and the phosphorylated histidine affect the p*K*_a_ of the lysine residues, and thereby its reactivity,^81^ we performed p*K*_a_ calculations based on MD-derived structures (see Supporting Materials and Methods). We find that the interactions between the coiled-coil core indeed polarize the nearby lysine 9, resulting in a decrease of the p*K*_a_ from ∼10 to as low as ∼7 (Figure 4C, Supporting Figure 18–25). Consequently, coiled-coil formation is expected to transiently populate a deprotonated state of lysine 9 on peptide B, potentially enabling it to function as a general Brønsted base during (Supporting Figure 14).

Using the spatial arrangement of lysine 9 and nearby water molecules obtained from our MD simulations, we further probed the energetics and putative dephosphorylation mechanism by performing density functional theory (DFT) calculations of the key lysines surrounding the phosphorylated histidine in the coiled-coil (see Supporting Materials and Methods, Supporting Tables 5 and 6). The DFT models suggest that the hydrolysis reaction proceeds via a proton-transfer mechanism mediated by intervening water molecules. First, the deprotonated lysine 9 of peptide B, reprotonates by extracting a proton from a neighboring water molecule, resulting in the formation of a hydroxide ion that, by an S_N_2-type nucleophilic attack on the phosphate, cleaves the P–N bond and releases inorganic phosphate. The reaction is coupled to a synchronous proton transfer from lysine 16 of peptide B to histidine 10 of peptide A, completing the dephosphorylation process. The reaction has a free energy barrier of around 21 kcal mol^–1^, which is in good overall agreement with the experimental rate of 0.27 h^–1^ (Δ*G* ^‡^ = 22.8 kcal mol^–1^ based on transition state theory). The reaction is exergonic by a few kcal mol^–1^ (Figure 4D), suggesting it is both kinetically and thermodynamically favored. Taken together, these data support the idea that cooperative electrostatic interactions favor folding into a coiled-coil in the active state, thereby catalyzing proton-transfer reactions and promoting dephosphorylation of peptide A.

## Conclusion and Outlook

The combined results have established a minimal, enzyme-free route to control dynamic phosphorylation of a peptide, which, in turn, regulates a protein–protein interaction motif through coiled coils. By engineering a single histidine residue into one helix, we created a switchable site whose charge inversion upon phosphorylation modulates both the thermodynamic stability and the kinetics of coiled-coil formation. We opted for a coiled-coil motif and phosphorylation, both of which are ubiquitous in biology. The design could thus offer a model for dynamic phosphorylation in biology, but also aid in making protein designs dynamic. Beyond phosphorylation-driven equilibrium stabilization, we found that the system is kinetically gated—the mechanical state of the system (bound vs unbound) feeds back on the reaction cycle, accelerating both activation and deactivation when peptide A is engaged with peptide B. Kinetic gating leads to the cycle operating with a slight clockwise bias. As the system is in a nonequilibrium steady state, the clockwise flux is on average 4.3 times greater than the counterclockwise flux, meaning four out of five cycles run clockwise and one runs counterclockwise. This demonstrates that the cycle is performing work. To do so more efficiently, we should increase the bias further. On one hand, to maximize the stored energy, the fuel-to-waste reaction would have to be faster than the kinetics of the equilibrium steps.^47^ To accelerate the reaction, a phosphate shuttle, like pyridine, could be added to the system, but the challenge would be to counterbalance the faster dephosphorylation.^56^ On the other hand, the activation kinetics would be faster in the unbound than the bound state (which is currently the opposite). To achieve this, we could use a phosphorylating agent bulkier than MAP. Alternatively, peptide B could be immobilized and physically separated from peptide A. For example, it could be cholesterol-modified and immobilized on a membrane while the fuel is supplied diffusively. That way, peptide A has to diffuse to the fuel source to activate, then diffuse to the membrane, where it forms a coiled-coil with peptide B to deactivate. In this scenario, the ratcheted cycle is converted into a pump in which molecule A is pumped back and forth.

While the present system is not yet a pump or motor, it demonstrates a design in which a reaction cycles gate coiled-coil lifetimes and composition using minimal molecular complexity. More broadly, it bridges protein design and systems chemistry by showing that a ubiquitous protein–protein interaction motif can be driven out of equilibrium by enzyme-free phosphorylation, providing a foundation for fuel-regulated interaction networks and future protein-based machinery.

## Supplementary Material















## Notes

Materials and Methods description and additional data; synthetic protocols and analysis; sample preparation; MD simulations and DFT calculations; kinetic model; ratcheting constant; NMR and HPLC analysis; CD and ITC analysis (PDF)
MD simulations of coils A and B (MP4)
MD simulations of coils A and B (MP4)
MD simulations of phosphorylated coil A and coil B (MP4)
MD simulations of phosphorylated coil A and coil B (MP4)
QM simulation of the dephosphorylation (MP4)
The code for analyzing the CD spectra and melting curves, as well as the kinetic model (XLSX)

## ∇

Department of Molecular and Cell Biology, University of California, Berkeley, 300F Li Ka Shing Center, Berkeley, CA 94720, United States

## Abbreviations

CC: coiled-coil
ATP: adenosine triphosphate
PPI: protein–protein interaction
MAP: monoamidophosphate
MD: molecular dynamics
DTF: density functional theory
CD: circular dichroism
HSQC: heteronuclear single quantum coherence
NMR: nuclear magnetic resonance
HPLC: high-performance liquid chromatography
ITC: isothermal titration calorimetry
HEPES: 4-(2-hydroxyethyl)-1-piperazineethanesulfonic acid
MRE: mean residual ellipticity
