# Supplementary material for: Enzyme-Free Phosphorylation with Kinetic Gating in a De Novo Coiled-Coil System
Source: J Am Chem Soc. 2026 May 19;148(21):21725–36. doi: 10.1021/jacs.6c02162 (PMC13244449; doi:10.1021/jacs.6c02162)
Supplement: Supplementary file 1 [file ja6c02162_si_001.pdf]

Supporting Information for:

Enzyme-free Phosphorylation with Kinetic Gating in a De Novo Coiled-coil system

Simone M. Poprawa,[1] Niklas L. Hoja,[2] Clara Hipp,[1†] Reece O. King,[3] Héctor Soria-Carrera,[1] Derek N. Woolfson,[3,4,5,6] Michael Sattler,[1] Ville R. I. Kaila,[2] Job Boekhoven[1]*

[1] Department of Bioscience, School of Natural Sciences, Technical University of Munich, Lichtenbergstrasse 4, 85748 Garching, Germany.

[2] Department of Biochemistry and Biophysics, Stockholm University, Svante Arrhenius väg 16C, 10691 Stockholm, Sweden.

[3] School of Chemistry, University of Bristol, Bristol BS8 ITS, United Kingdom

[4]Max Planck-Bristol Centre for Minimal Biology, School of Chemistry, University of Bristol, Bristol BS8 ITS, United Kingdom

[5] School of Biochemistry, University of Bristol, Bristol BS8 ITD, United Kingdom

[6] Center for Protein Design, University of Copenhagen, DK-2100 Copenhagen, Denmark

Table of Contents

I. Supporting Materials and Methods 3

A) Materials 3

B) Methods 6

C) Kinetic model 12

D) K_r for biomolecular process 14

II. Supporting Tables 16

III. Supporting Figures 24

I. Supporting Materials and Methods

(A) Materials

All chemicals were purchased from Sigma-Aldrich, VWR, or TCI Europe and used without further purification. General solvents were purchased from Sigma Aldrich in analytical or synthesis grade and used without further purification. Fmoc-Rink-Amide-TentaGel XV was purchased from Iris Biotech. All peptides were synthesized by standard Fluoren-9-ylmethoxycarbonyl (Fmoc) solid-phase peptide synthesis as described below.

Synthesis of potassium mono-amidophosphate. MAP was synthesized, as previously reported by Ying-Fei Wei and Harry R. Matthews.[1] Into 75.0 mL ice-cold 10% ammonium hydroxide solution 5.00 g phosphorus(V)oxychloride (32.6 mmol) were added dropwise over 20 min and stirred for 15 min on ice. 250 mL acetone were added and then stirred for five more minutes at room temperature (RT). The mixture was transferred to a separation funnel, and after 5 min the phases were separated, and the bottom one was collected. A white precipitate crystallized while the collected water phase was acidified with 2.00 mL acetic acid to pH 6. The mixture was stored in the fridge (4 °C) for 20 min to facilitate crystallization and 20 min at RT. The ammonium monoamidophosphate was filtered off and washed with ice-cold 10.0 mL absolute EtOH and 5.00 mL diethyl ether. It was dried overnight in the desiccator, and 0.582 g $NH_4PO_3NH_2$ was obtained (5.10 mmol, 16%). The ammonium monoamidophosphate was dissolved in 2.00 mL 50 % KOH (w/v) solution and heated for 10 min at 60 °C. The solution was cooled to 5 – 10 °C in a water bath and acidified with 0.75 mL acetic acid to pH 6 with acetic acid. The mixture was added to 100 mL of absolute EtOH and crystallized for 1.5 h at RT. Potassium monoamidophosphate was filtered off. It was later washed with ice-cold 5.00 mL absolute EtOH and 4.00 mL diethyl ether and dried in the desiccator overnight. 0.596 g MAP were obtained (4.41 mmol, 86 %). It was characterized by ^{31}P-NMR and elemental analysis.

^{31}P-NMR (D_2O, pH = 6.5, 500 MHz): δ -3.88

Elemental analysis:

theoretical values: H: 2.24%; N: 10.37%; K: 28.94%; O: 35.53%; P: 22.93%

measured values: H: 2.26%; N: 10.22%; K: 29.3%; P: 22.31%.

Microwave-assisted, automated solid phase peptide synthesis. The peptides were synthesized in 0.05 mmol scales on a peptide synthesizer Liberty Blue® from CEM. A high-swelling Fmoc-Rink-Amide-TentaGel XV (0.05 mmol, 100-200 mesh, 0.24 mmol/g) was used to synthesise the amidated peptides. Amino acid couplings were performed with the following sequence: Fmoc-deprotection was achieved by reacting 4 mL of a piperidine solution in DMF (20 v/v%) with the resin for 30 seconds at 75°C. The deprotection was repeated for 3 minutes at 75°C with a washing step with DMF in between. 4 eq. of the corresponding Fmoc-protected amino acid, 4 eq. of DIC in DMF and 4 eq. ethyl (hydroxyimino)cyanoacetate (Oxyma) in DMF were reacted with the resin. The reaction mixture was heated to 25°C for 2 minutes followed by 8 minutes at 50°C in the microwave. The reaction mixture was heated to 25°C for 25 minutes for arginine couplings. Each amino acid coupling was repeated one time and the resin was washed with DMF in between. After the last coupling and deprotection, the peptide was acetyl end-capped in a peptide reaction vessel at room temperature. The resin was washed with DMF, and 6 eq. of acetic anhydride and 6 eq. of DIPEA in DMF solution were added to the resin and agitated for 3 h at RT. The resin was washed with DMF and DCM. Sidechain deprotection and final cleavage from the resin was achieved by reacting the resin with a solution of 2.5% water, 2.5% triisopropylsilane (TIPS) and 95% trifluoroacetic acid (TFA) for 2 h under continuous agitation at RT. The solution was collected and the resin was washed with DCM. Solvents were removed by evaporation in N_2 flow. The crude peptide was precipitated with ice-cold diethyl ether and kept at -20°C for 1 h. The precipitate was spun down and the ether removed. The crude peptides were dissolved in H_2O:ACN (70:30) and purified using a reversed-phase preparative HPLC (Thermo Fisher Dionex Ultimate 3000, Hypersil Gold 250 × 4.8 mm, Thermo ScientificTM DionexTM ChromelonTM Chromatography Data System, eluted with 10 mM NH_3HCO_3 water:ACN for A peptides; RP-HPLC on an Agilent 1260 Infinity ll setup, Agilent InfinityLab ZORBAX SB-C_{18} column 250 mm x 21.2 mm, 5 µm particle size, eluted with 0.1% TFA H_2O:ACN for B peptides). A linear gradient was used for the purification, and the different fractions were collected at 220 nm.

All peptides were lyophilized (Christ Freeze Dryer Alpha 2-4 LDplus, VWR) and stored at -20 °C. They were characterized by electrospray ionization mass spectrometry (ESI-MS, Thermo Scientific, LCQ Fleet ION Trap Mass Spectrometer) in positive mode and analytical HPLC (Vanquish DUO HPLC system (Chromeleon software version 7.2.10 ES) with a Hypersil-Gold, reversed-phase C_{18} column (particle size: 3 µm, length: 100 mm, ID: 2.1 mm), eluted with a gradient of 0.1% TFA H_2O:ACN and detected at 220 nm.

Synthesis and purification of activated, phosphorylated peptide A. To phosphorylate peptide A, we prepared a roughly 1.8 mM peptide A solution of the purified and lyophilized fraction in 3 mL 10 mM NH_3HCO_3 and added 100 mM MAP and let it react at 25°C for 3 days. The reaction solution was

purified using a reversed-phase preparative HPLC (Thermo Fisher Dionex Ultimate 3000, Hypersil Gold 250 × 4.8 mm, Thermo ScientificTM DionexTM ChromelonTM Chromatography Data System, eluted with 10 mM NH_3HCO_3 water:ACN). A linear gradient was used for the purification, and the different fractions were collected at 220 nm.

(B) Methods

Sample preparation. We prepared stock solutions of the peptides by dissolving them in 15 mM HEPES buffer pH 6.5, after which we readjusted the pH to pH 6.5 with KOH or HCl. Stock solutions of MAP were prepared by dissolving the MAP powder in 15 mM HEPES buffer pH 6.5. We prepared the stock solutions of 0.5 M MAP freshly. We prepared stock solutions of the activated peptide A by dissolving them in 15 mM HEPES buffer pH 6.5, after which we readjusted the pH to pH 6.5 with KOH or HCl. Due to hydrolysis occurring, the concentration of activated peptide A is lower than that determined using µDrop plate and a plate reader. The stock solution of activated peptide A was freshly prepared and stored in the freezer until use.

For the standard condition experiments, samples consisted of 50 µM peptide A in the presence and absence of 50 µM peptide B, in a 15 mM HEPES pH 6.5 buffered solution. Reaction cycles were initiated by adding the high-concentration MAP to the reaction solution to obtain a final concentration of 12.5 mM. We carried out all experiments at 25 (±0.5)ºC.

To investigate the catalytic activity of the side-chain residues, we prepared 10 M stocks of potassium acetate, acetamide, and ethylenediamine in 15 mM HEPES 6.5. We adjusted the pH to 6.5 with HCl or KOH and recalculated the concentrations of the stock solutions.

The samples with the side-chain mimics and MAP added from the beginning (Fig. 3C) contained 50 µM peptide A, 1 M side-chain mimics, in a 15 mM HEPES, pH 6.5, solution with 2.6 M ionic strength. Reaction cycles were initiated by adding the high-concentration MAP to the reaction solution to obtain a final concentration of 12.5 mM. We carried out all experiments at 25 (±0.5)ºC.

The samples with activated peptide A and side-chain mimics added from the beginning (Fig. 3D) contained 50 µM activated peptide A in a 15 mM HEPES, pH 6.5, solution with 2.6 M ionic strength. We added the high-concentration side-chain mimic stock solutions to obtain a final concentration of 1 M. We carried out all experiments at 25 (±0.5)ºC.

Kinetic model. We used a kinetic model for predicting the evolution of the reaction network over time. The kinetic model chapter provides a brief description of the model. The rate constants we used in this work are given in Supporting Tables 3 and 4.

Elemental analysis. The CHNS values are determined simultaneously by combustion analysis in a EuroEA Elemental Analyser from HEKAtech. The following standard substances were included in the analyses: 2x BBOT and 1x chloro-2,4-dinitrobenzene. An error tolerance range of ± 0.3% can be

specified. Potassium was determined using acid digestion and subsequent measurement on an Agilent 280 FS-AA atomic absorption spectrometer (flame AAS). Potassium dihydrogen phosphate was used as the reference substance. The error tolerance range is ± 0.5%. Phosphorus was determined after acid digestion and subsequent measurement on a Cary100 UV/VIS photometer from Agilent. Triphenylphosphine was used as the test substance. The error tolerance range is ± 0.3%. As obtained from the central analytics of the Technical University of Munich.

^{31}P-NMR spectroscopy kinetic measurements. Over time, the fuel consumption was quantitatively monitored with ^{31}P-NMR on a Bruker AV500CR NMR-spectrometer. An inverse gated H-decoupled[2] method with 16 scans and 25 s relaxation delay was used. As internal standard, phosphonoacetic acid (10 mM, δ = 15.7 ppm[3]) was used. The concentrations were calculated by using the following equation.[2]

$$c_{\mathrm{x}} = c_{\mathrm{standard}} \times \frac{\mathrm{integral_x}}{\mathrm{integral_{standard}}} \qquad \text{(Supporting Eq. 1)}$$

With c_{x}: concentration of compound x, c_{standard}: concentration of the internal standard, $\mathrm{integral_x}$: integral of compound x, $\mathrm{integral_{standard}}$: integral of internal standard.

For ^{31}P-NMR consumption of fuel measurements, 100 µL of the samples were prepared. The samples mixture was prepared as described above. Additionally, to get the hydrolysis of MAP under the used conditions a sample only consisting of 12.5 mM was prepared. The hydrolysis of MAP and fuel consumption, respectively, were determined by applying Supporting Eq. 1. The NMR spectra were analyzed using MestReNova© software (version 14.2.3-29241).

Circular dichroism spectroscopy (CD-spectroscopy). Measurements were performed on a Jasco J-1500 CD Spectrometer. Samples were prepared as described above and transferred into a 1 mm quartz cell (Hellma Analytics) cuvette. The CD spectra measurements were performed in the wavelength range between 200 nm and 260 nm with a scanning speed of 200 nm min^{-1}, 1 nm steps, a bandwidth of 1 nm. 3 accumulations were performed for each measurement. For the spectra measurement over time to monitor the change in helicity after the addition of MAP, the CD spectra were recorded every 15 min with a scanning speed of 200 nm min^{-1}, 1 nm steps, and a bandwidth of 1 nm. 5 accumulations were performed for each measurement. We calculated the MRE using an adapted version of rstoolbox.[4]

After stopping the measurement 3 melting curves were performed: the first one from 5-90°C, the second one reverse from 90-5°C and the third one from 5-90°C. The third one was used to determine the minimum and maximum MRE and thus fraction bound and delta fraction bound for each reaction.

The melting curves were performed at 222 nm in the temperature range from 5 to 90°C with the holder as control sensor, a ramp rate of 1°C min-1, waiting 16 sec before acquiring data, and a bandwidth of 1 nm. We calculated the melting temperature using an adapted version of rstoolbox.[4]

The code used to analyse the raw data of the spectra and melting curves can be found here:

https://github.com/sim1poprawa/CC-phosphorylation

UV/Vis spectroscopy. The UV/Vis measurements to determine the concentration of the peptide stock solutions were carried out using a Multiskan FC (ThermoFisher) microplate reader and a μDrop plate. For the concentration determination, the Technical Note "A theoretical and practical guide for spectrophotometric determination of protein concentrations at 280 nm" published by thermoscientific was adapted. 2.5 μL of buffer was used as a blank for every 2.5 μL peptide sample. The temperature (25 ± 0.5ºC) was set 10 min before starting the measurement. Spectra were recorded from 200 to 400 nm, every nm a data point was acquired.

The extinction coefficient for peptide B (Tyrosine) was 1280 $M^{-1}cm^{-1}$ and for peptide A (Tryptophan) was 5690 $M^{-1}cm^{-1}$.[5]

ESI-MS. An LCQ Fleet Ion Trap Mass Spectrometer (Thermo Scientific) was used for ESI-MS experiments. The data was evaluated using the Thermo Xcalibur Qual Browser 2.2 SP1.48 software. 5 μL of the peptide stocks were injected.

ITC measurements. ITC experiments were performed with a MicroCal PEAQ-ITC from Malvern Pananalytical. All experiments were performed at 25 °C. The following conditions were used: peptide A or phosphorylated A (in HEPES 15 mM, pH 6.5) was titrated with the peptide B (in 15 mM HEPES, pH 6.5): 19 injections, 2 μL each, concatenated for peptide A. A composite type control was performed by titrating the corresponding amount of peptide B in 15 mM HEPES buffer (pH 6.5) and used to correct for dilution enthalpy and by titration 15 mM HEPES in the corresponding amount of peptide A. Data were fitted to a single set of sites binding isotherm using the PEAQ-ITC Analysis software.

Analytical HPLC kinetic measurements. For the kinetic measurement of the transient, activated species of the peptide A, an analytical HPLC (Thermo Fisher Vanquish SINGLE HPLC system with EC 150/4 NUCLEODUR C_{18} Pyrmid, 3 μm (particle size: 3 μm, length: 150 mm, ID: 4 mm), Machery-Nagel; eluted with a linear gradient of NH_3HCO_3 10 mM and ACN at 30 °C column temperature) was used. The Chromeleon 7 Data System Software (Version 7.3.1) was used to evaluate the received data.

100 μL of samples were prepared as described above and the reaction solution was transferred to inserts for a 1.5 mL screw cap HPLC glass vial. 4 μL of the solution was injected without any further dilution.

Calibration curves for the peptide A (in buffer) were performed in triplicate with the corresponding method. The calibration factor of peptide A was used for activated peptide A. Retention times and calibration values are given in the table for the characterization of the precursors.

Protein expression and purification of $^{13}C^{15}N$ labelled peptides. The plasmids encoding the peptide sequences were cloned into a pETM11 vector containing a TEV cleavable His_6-ProteinA tag, and the recombinant protein was expressed in *E. coli* BL21 (DE3) cells in M9 minimal medium supplemented with 1 g/l $^{15}NH_4Cl$ or 1 g/l $^{15}NH_4Cl$ and 1 g/l ^{13}C-Glucose. The bacterial cells were grown at 37 °C to an OD_{600} of 0.8, subsequently induced with 1.0 mM IPTG, and the protein was expressed at 18 °C overnight. The cells were harvested (7808 × *g*) and resuspended (50 mM Tris, pH 8.0, 500 mM NaCl, 10 mM imidazole, supplemented with lysozyme, 1 mg/ml DNase, 2 mM $MgSO_4$, and protease inhibitor). After lysis of the cells using sonication and centrifugation (38759 × *g*, 1 h), the cleared lysate was added to Ni–NTA resin, washed with 2 M NaCl, and eluted with 500 mM imidazole. Peptides A and B were further purified by ion-exchange chromatography on a HiTrap Q column (Cytiva) (20 mM Tris, pH 8.0, gradient from 0 to 1 M NaCl in 10 column volumes) and a HiTrap SP column (20 mM Phos, pH 6.0, gradient from 0 to 1 M NaCl in 10 column volumes), respectively. This was followed by size-exclusion chromatography on a HiLoad 16/600 Superdex 75 column (GE Healthcare) (20 mM sodium phosphate, pH 6.5, 150 mM NaCl). The His_6-ProteinA tag was subsequently cleaved with His-tagged TEV protease (4°C, overnight), and the protein was purified on a second Ni–NTA column to remove the Tag and TEV protease.

Peptide	Vector backbone	Amino acid sequence
A	pETM11, with His-ProteinA tag-TEV	GAM G EIAALEK EHAALKW EIAALEQ GG
B	pETM11, with His-ProteinA tag-TEV	GAM G KIAALKY KNAALKK KIAALKQ GG

NMR spectroscopy ($^1H,^{15}N$ HSQC). All NMR samples were measured in NMR buffer (15 mM HEPES, pH 6.5) containing 10% (*v*/*v*) D_2O on 600 MHz, 950 MHz, and 1200-MHz Bruker Avance NMR spectrometers equipped with cryogenic triple-resonance gradient probes. All spectra were measured in 3 mm NMR tubes. $^{15}N^{13}C$-labeled A peptide (280 μM), and ^{15}N-labeled B peptide (150 μM) were mixed in an equimolar ratio with the synthesized B and A peptides, respectively, and $^1H,^{15}N$ HSQC spectra were measured at 5 °C. For temperature series, $^1H,^{15}N$ HSQC spectra of ^{15}N-labeled B peptide (150 μM) with an equimolar ratio with the synthesized A peptide were measured at 25 °C, 20 °C, 15 °C, 10 °C, and 5 °C. NMR spectra were processed with TOPSPIN3.5 (Bruker) and analyzed using NMRFAM-Sparky.[6]

Molecular dynamics (MD) simulations and pK_a calculations. MD simulations of peptides A and B were carried out based on AlphaFold3[7] predictions of the initial structure. Simulations were initiated

with the peptides initially placed in both parallel and antiparallel configurations, *ca.* 20 Å apart from each other, both with and without phosphorylation of His9^B (Table S1). The models were solvated with TIP3P water molecules[8], and neutralized with 0.10 M NaCl. Each system was minimized, followed by heating to T=310 K in an *NVT* ensemble, and equilibration under *NPT* conditions at T=310 K and p=1 atm, using a Langevin thermostat and barostat. Long-range electrostatic interactions were treated using the Particle Mesh Ewald (PME) method, with a grid separation of 1 Å, and describing the system using the CHARMM36 force field[9]. The system was simulated for 500 ns in four independent replicas for each condition using a 2 fs integration timestep. All MD simulations were performed using NAMD (versions 2.14 and 3.0)[10], while the systems were visualized using Visual Molecular Dynamics (VMD)[11].

pK_a values during MD simulations were analyzed based on Poisson-Boltzmann electrostatic (PBE) calculations with Monte Carlo (MC) sampling of the protonation space. To this end, the linearized PBE was solved based on structures extracted from the MD simulations, with solvation energies and electrostatic interactions computed using the adapted Poisson-Boltzmann solver (APBS).[12] The protein was treated using explicit point charges, embedded in a polarizable dielectric medium for the protein surroundings (ε=4) and bulk water (ε=80). Protonation states we computed based on MC sampling, as implemented in an in-house extension of Karlsberg2[13].

DFT calculations. The energetics of the phosphate hydrolysis reaction was explored based on quantum chemical density functional theory (DFT) calculations. To this end, models were constructed based on the MD simulations of the phosphorylated coiled-coil system $A_{Phos}B$ in the parallel orientation (Fig. X, B). The DFT models, comprised 124 atoms, including the $His_{Phos}10^A$, Lys9^B, Lys14^A, Lys16^B, Glu7^A, and 15 adjacent water molecules. The amino acid residues were cut at the Cα-Cβ bond, and saturated with hydrogen atoms. The structures were optimized at the B3LYP-D3/def2-SVP/ε=80 level[14-18], with the Cβ atoms fixed during the structure optimization to preserve the backbone orientation. The structure of the reactant (His-P_i) and hydrolyzed product states (His+HPO_3^-) were optimized, followed by transition state optimization. Single-point electronic energy calculations were performed using a def2-TZVP basis sets (13-14) at the ωB97X-D (12), M06-2X/D3 (11), TSSPh/D3 (10), and B3LYP/D3 levels. The reaction free energies were computed with vibrational and zero-point energy corrections based on calculation of the numerical calculation of the molecular Hessian. All DFT calculations were carried out with TURBOMOLE (version 7.7.0).[19]

(C) Kinetic model

A kinetic model was written to describe the evolution of the species and their concentration involved in the chemical reaction network. The concentrations of each reactant were calculated for every 5 minutes in the cycle. The model was used to obtain fitted curves based on ^{31}P-NMR or HPLC data that described the evolution of the concentration of the phosphorylated species, fuel, and waste.

The reaction rate constant of the hydrolysis of pHis was determined empirically when no fuel was present anymore by using the purified pHis. Therefore, the predicted and the experimentally measured evolution fit nicely. The hydrolysis of MAP, as well as the reaction rate constant of the hydrolysis in the presence of peptide B, were calculated empirically, but the other constants were fitted and thus might show higher deviations. Despite that, the fits align very well with the experimental data.

The kinetic model is suitable for predicting the evolution of a single species and can potentially aid in designing a self-assembling system. But the reaction rate constants, which are not determined by the data, should not be compared directly.

Reaction 0 (k_0):

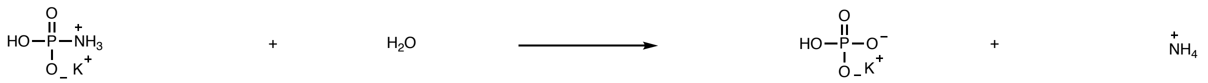

The direct hydrolysis of the fuel (MAP) to inorganic phosphate follows pseudo-first-order kinetics with respect to MAP. The rate constants and the corresponding half-lives are summarized in Supporting Tables 3 and 4.

Reaction 1 (k_1 and k_2):

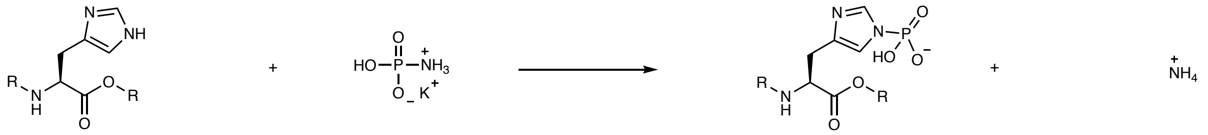

The reaction of MAP with the His-modified peptide A to the pHis-product follows second-order kinetics. We assumed that the signal of the phosphorylated species, observed with analytical HPLC (Supporting Figures 8 and 10), corresponded to the 3-phosphohistidine isomer, as it is the most stable and longest-lasting isomer.[20] In the absence of peptide B, the reaction rate consists of one term following the second-order kinetics (k_1). In the presence of peptide B, the reaction rate consists of two terms: the aforementioned one, with the reaction rate constant in the absence of peptide B (k_1), and the

term in the presence of peptide B (k_2). In the presence of peptide B, the ratio of peptide A for the first (disassembled) and the second term (assembled to the coiled-coil state) is determined by the melting curve of peptide A and B at 25°C. Reaction rate constant k_1 is determined empirically by following the formation of the phosphorylated species with analytical HPLC and fitting the model to the concentration profile. The reaction rate constant k_2 is empirically set to be 30 times faster than k_1. The rate constants are shown in Supporting Tables 3 and 4

Reaction 2 (k_{-1} and k_{-2})

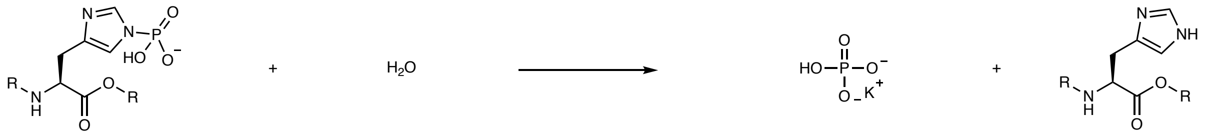

The hydrolysis of pHis follows pseudo-first-order kinetics with respect to pHis. Reaction rate constant k_{-1} is determined experimentally by following the hydrolysis of the phosphorylated species with analytical HPLC after the fuel was consumed. The reaction rate constant k_{-2} is determined by adding peptide B to the purified, activated peptide A and monitoring the hydrolysis of the activated species with analytical HPLC. The rate constants are shown in Supporting Tables 3 and 4.

(D) K_r for biomolecular process

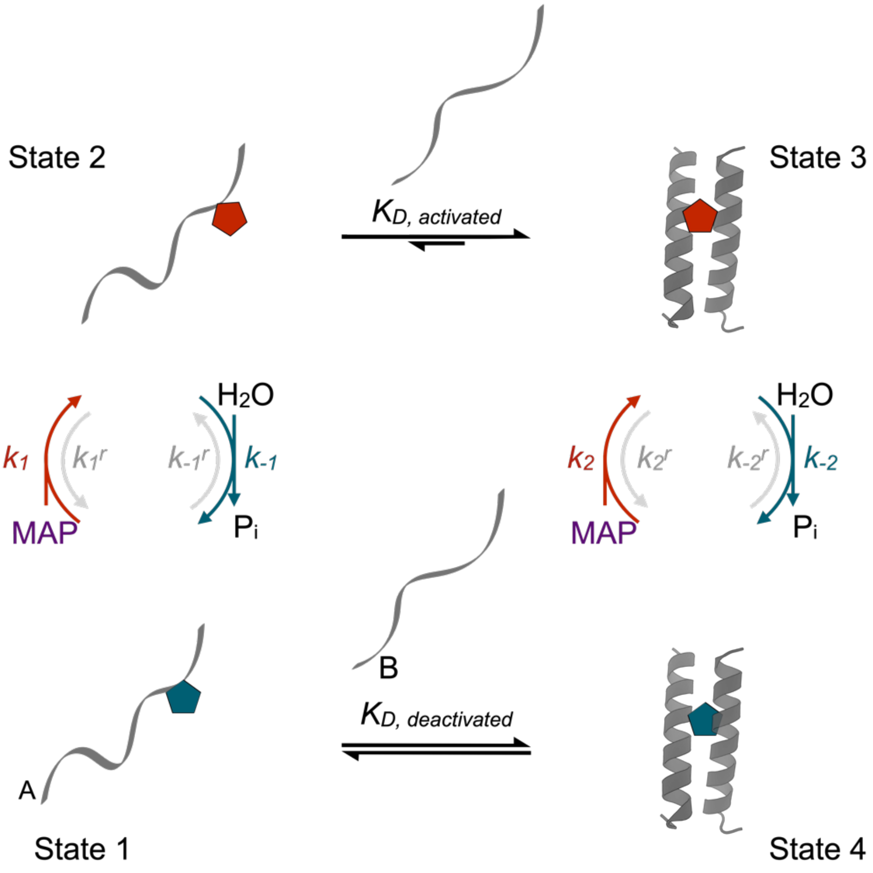

The reaction rate constants k_1 and k_{-1} have their reverse constants k_1^r and k_{-1}^r. The reverse reaction rates are negligible ($k_n^r \approx 0$), as their forward reactions can be assumed to be irreversible (the fuel is “irreversibly” consumed). Thus $k_1 + k_1^r \approx k_1$ and $k_{-1} + k_{-1}^r \approx k_{-1}$. The same is valid for k2 and k-2. K_r is an expression of the directionality, the directional bias, along a certain cycle with respect to a specific moiety. This is not an unimolecular self-assembly process, so we decided to focus on peptide a as the specific moiety. The cyclic pathways we compared were state 1 → state 2 → state 3 → state 4 → state 1 and its reverse state 1 → state 4 → state 3 → state 2 → state 1. The availability of peptide B to assemble in the activated or the deactivated state is the same for both and doesn’t influence the ratio of the state. Therefore, the direction is not dependent on concentrations, and all states have the same bias.

Thus, following equation for directionality Kr by Ragazzon and Prins[21] can be adjusted to Supporting Eq. 2. With $k_n^r \approx 0$, formally leading to the equation for kinetic asymmetry q (Supporting Eq. 3).

$$K_r = \left(\frac{k_{1Ff} + k_{1Wf}}{k_{1Fb} + k_{1Wb}}\right)^n K_2 \left(\frac{k_{3Ff} + k_{3Wf}}{k_{3Fb} + k_{3Wb}}\right)^{-1} K_4^{-1} \text{ [21]}$$

$$K_r = \frac{\frac{k_1 + k_{-1}^r}{k_{-1} + k_1^r} \times \frac{[\text{peptide B}]}{K_{D,activated}}}{\frac{k_2 + k_{-2}^r}{k_{-2} + k_2^r} \times \frac{[\text{peptide B}]}{K_{D,deactivated}}} \qquad \text{Supporting Eq. 2}$$

$$K_r \approx q = \frac{\frac{k_1}{k_{-1}} \times \frac{[\text{peptide B}]}{K_{D,activated}}}{\frac{k_2}{k_{-2}} \times \frac{[\text{peptide B}]}{K_{D,deactivated}}} = \frac{k_1 \times k_{-2} \times K_{D,deactivated}}{k_2 \times k_{-1} \times K_{D,activated}} \qquad \text{Supporting Eq. 3}$$

II. Supporting Tables

Supporting Table 1. Characterization of peptides (all of them are acetylated and amidated).

Peptide A1		M_W calculated [g/mol]	2576.85
GEIAALEKEHAALEWEIAALEQGG		m/z	2577.4 $[M+H]^+$

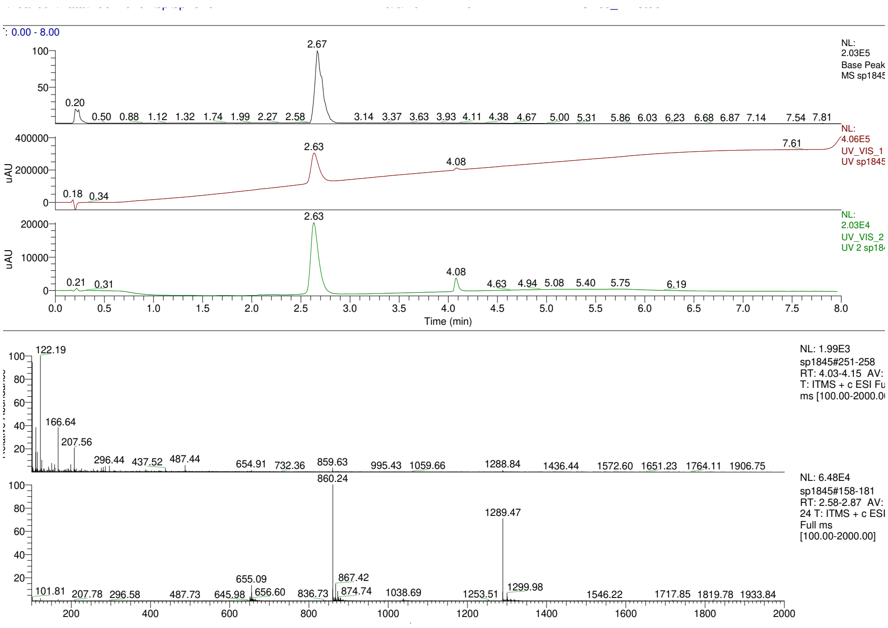

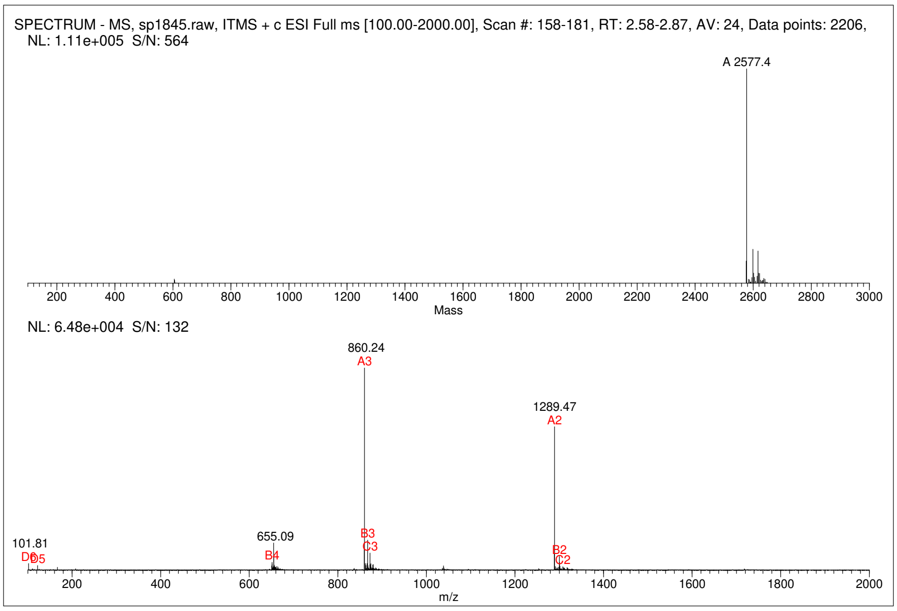

Peptide A<br><br>GEIAALEKEHAALKWEIAALEQGG	M_W calculated [g/mol]	2575.91
	m/z	2576.1 [M-H]$^+$
	Retention time [min]	16.5
	(10 mM NH_3HCO_3 H_2O:ACN)	(Calibration factor = 0.2045)

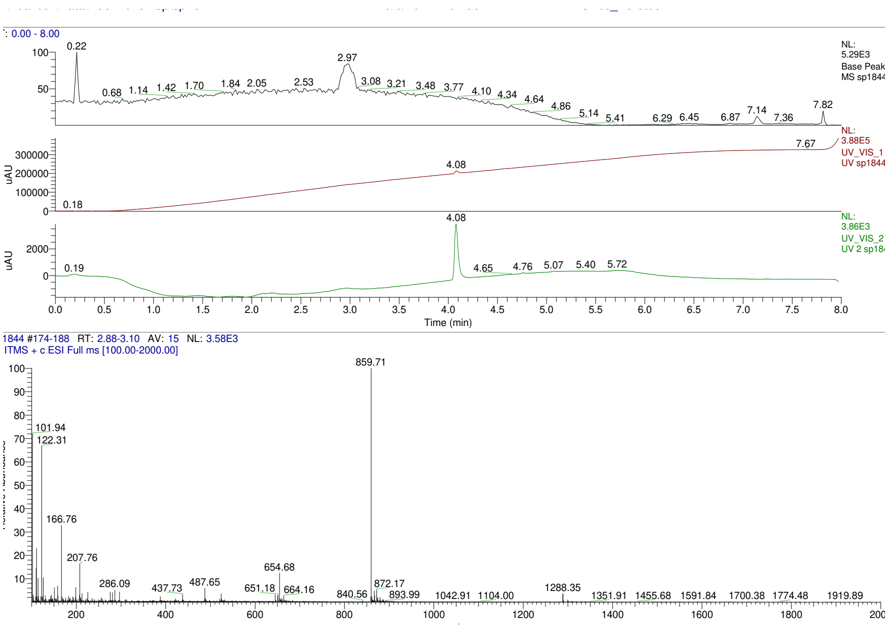

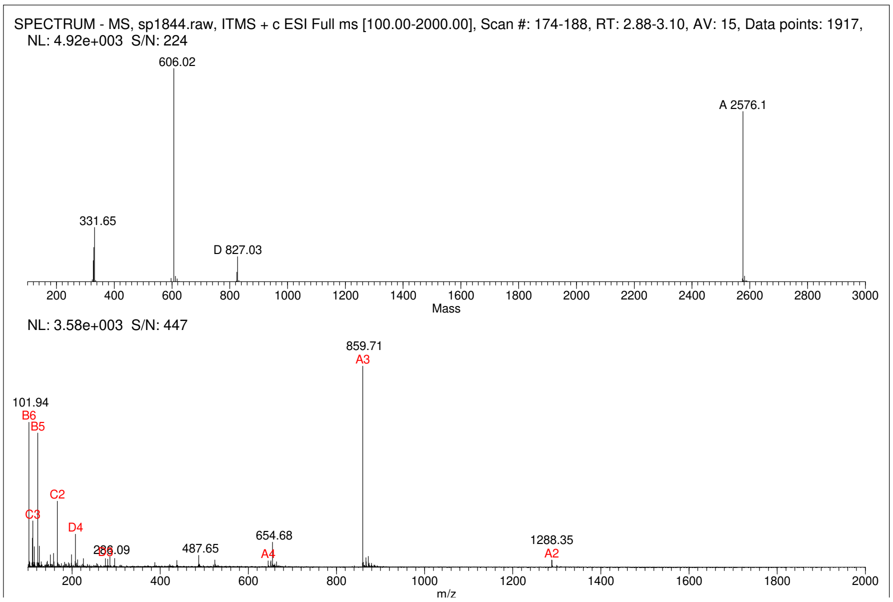

Peptide B1	M_W calculated [g/mol]	2524.19
GKIAALKYKIAALKKKIAALKQGG	m/z	2525.5 $[M-H]^+$

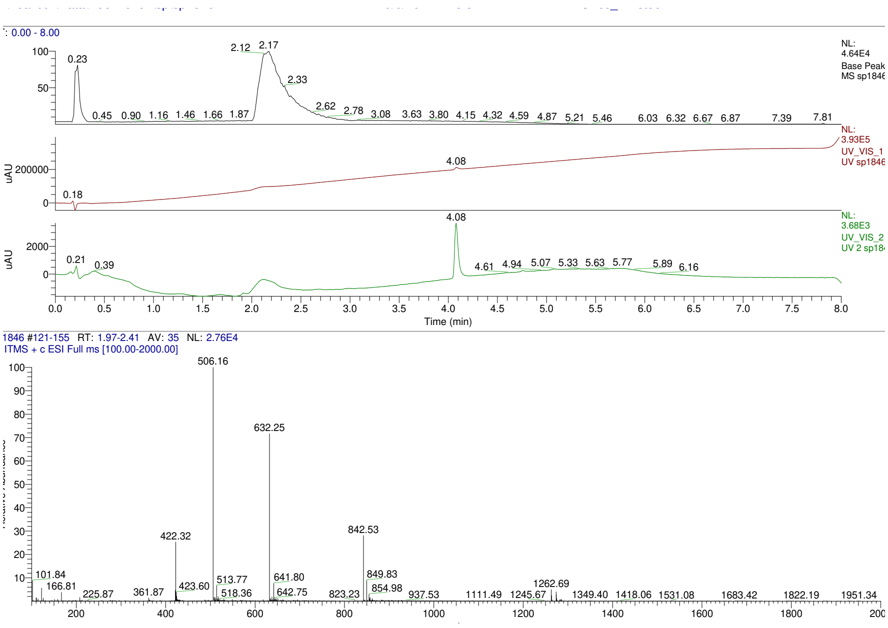

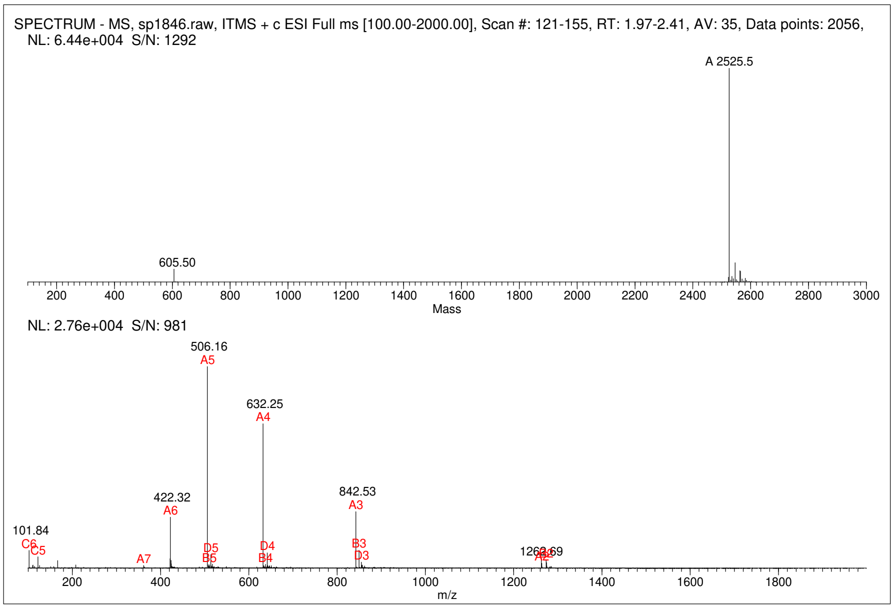

Peptide B	M_W calculated [g/mol]	2525.13
GKIAALKYKNAALKKKIAALKQGG	m/z	2525.2 [M]

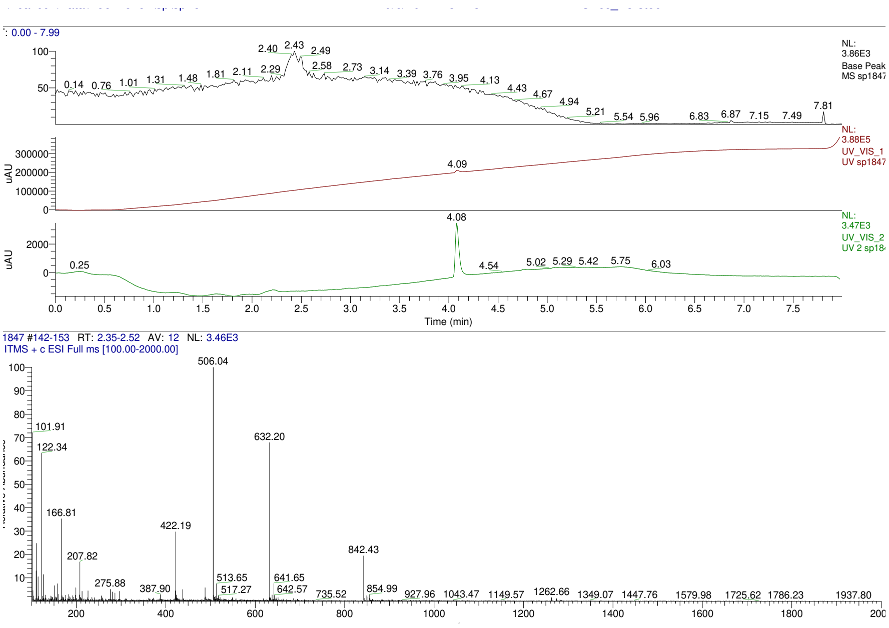

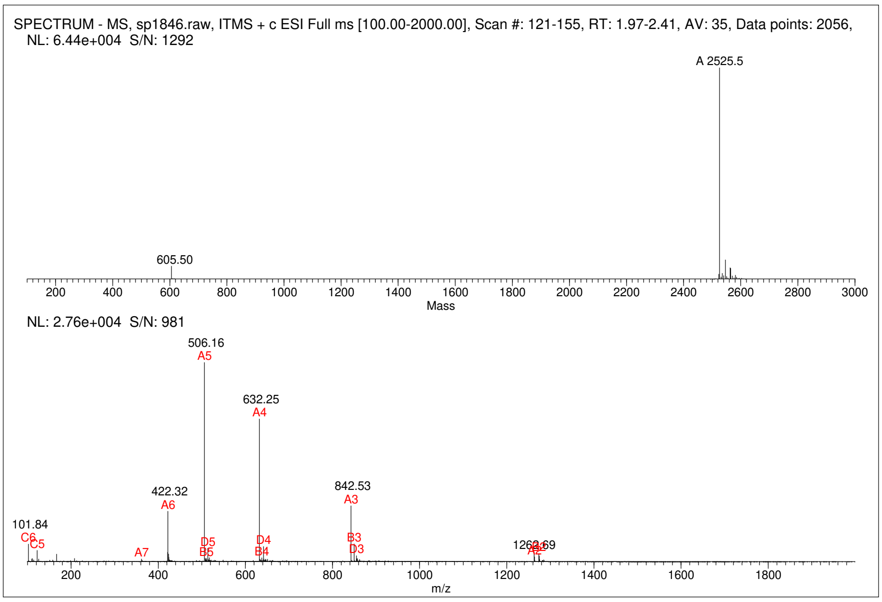

Supporting Table 2. List of MD simulations. All lysine residues were modeled in the protonated (cationic) state, and glutamate residues in the deprotonated (anionic) state.

Simulation	Time (ns)	Modelled State	Dimensions (Å)	*N* atoms
A	4 x 500	Peptide A,<br>His: N_δ protonated	80 x 65 x 85	53,600
A_{Phos}	4 x 500	Peptide A,<br>His: N_ε phosphorylated PO_3^{2-}	80 x 65 x 85	53,600
AB	4 x 500	Peptide A and B,<br>parallel orientation<br>His: N_δ protonated	100 x 75 X 95	79,100
AB ap	4 x 500	Peptide A and B,<br>antiparallel orientation<br>His: N_δ protonated	100 x 75 x 95	79,100
A_{Phos}B	4 x 500	Peptide A and B,<br>parallel orientation,<br>His: N_ε phosphorylated PO_3^{2-}	100 x 75 x 95	81,500
A_{Phos}B ap	4 x 500	Peptide A and B,<br>antiparallel orientation<br>His: N_ε phosphorylated PO_3^{2-}	100 x 75 x 95	81,500
**Total**	**12 µs**			

Supporting Table 3. Apparent hydrolysis of MAP in in the presence and absence of 50 µM peptide A and/or peptide B in a 15 mM HEPES pH6.5 buffered solution. The error is the average of the absolute deviations of constants from their mean (n = 3).

Conditions	Apparent hydrolysis constant of MAP [h^{-1}]
Pure MAP	$(5.90 \pm 0.76) \times 10^{-2}$
50 µM A	$(5.34 \pm 1.0) \times 10^{-2}$
50 µM A<br>50 µM B	$(3.88 \pm 1.2) \times 10^{-2}$

Supporting Table 4. Rate constants used in the kinetic model for the cycle with peptide A in the presence and/or absence of peptide B.

	Reaction rate constants
k_1 [mM^{-1} h^{-1}]	2.50×10^{-4}
k_{-1} [h^{-1}]	$(1.10 \pm 0.1) \times 10^{-2}$ [a]
k_2 [mM^{-1} h^{-1}]	$k_1 * 30 = 7.5 \times 10^{-3}$
k_{-2} [h^{-1}]	$(2.67 \pm 0.03) \times 10^{-1}$ [a]

[a] empirically determined and error is the average of the absolute deviations of constants from their mean (n = 3).

Supporting Table 5. Overview of DFT calculations. All DFT models were constructed from snapshots of the $A_{Phos}B$ parallel extracted from the MD simulation. Each model contained $His_{Phos}10^A$, $Lys9^B$, $Lys14^A$, $Lys16^B$, $Glu7^A$, and 15 explicit water molecules, resulting in N = 124 atoms.

Calculation	Dielectric Constant (ε)	Theory level	Comment
**Q1**	4	B3LYP-D3/ def2-SVP	Geometry optimization, vibrational/ZPE calculations
**Q2**	35	B3LYP-D3/ def2-SVP	Geometry optimization, vibrational/ZPE calculations
**Q3**	80	B3LYP-D3/ def2-SVP	Geometry optimization, vibrational/ZPE calculations
**Q4**	4	B3LYP-D3/ def2-TZVP	Single point energy for calculations for optimized geometry from Q1
**Q5**	35	B3LYP-D3/ def2-TZVP	Single point energy calculations for optimized geometry from Q2
**Q6**	80	B3LYP-D3/ def2-TZVP	Single point energy calculations for optimized geometry from Q3
**Q7**	80	MO6-2X/ def2-TZVP	Single point energy calculations for optimized geometry from Q3
**Q8**	80	TPSSh-D3/ def2-TZVP	Single point energy calculations for optimized geometry from Q3
**Q9**	80	ωB97X-D/ def2-TZVP	Single point energy calculations for optimized geometry from Q3

Supporting Table 6. Benchmarking the dephosphorylation energetics. The model system comprised N=124 atoms (Supporting Figure 15). Electronic energies were computed at the def2-TZVP/ε=80 (DFT) level.

**State**	**B3LYP-D3 (Q7)**	**MO6-2X-D3 (Q8)**	**TPSSh-D3 (Q9)**	**ωB97X-D (Q10)**
**Reactant**	0.0	0.0	0.0	0.0
**Transition**	26.2	22.8	20.0	27.5
**Product**	-2.5	-4.6	-4.7	-2.9

III. Supporting Figures

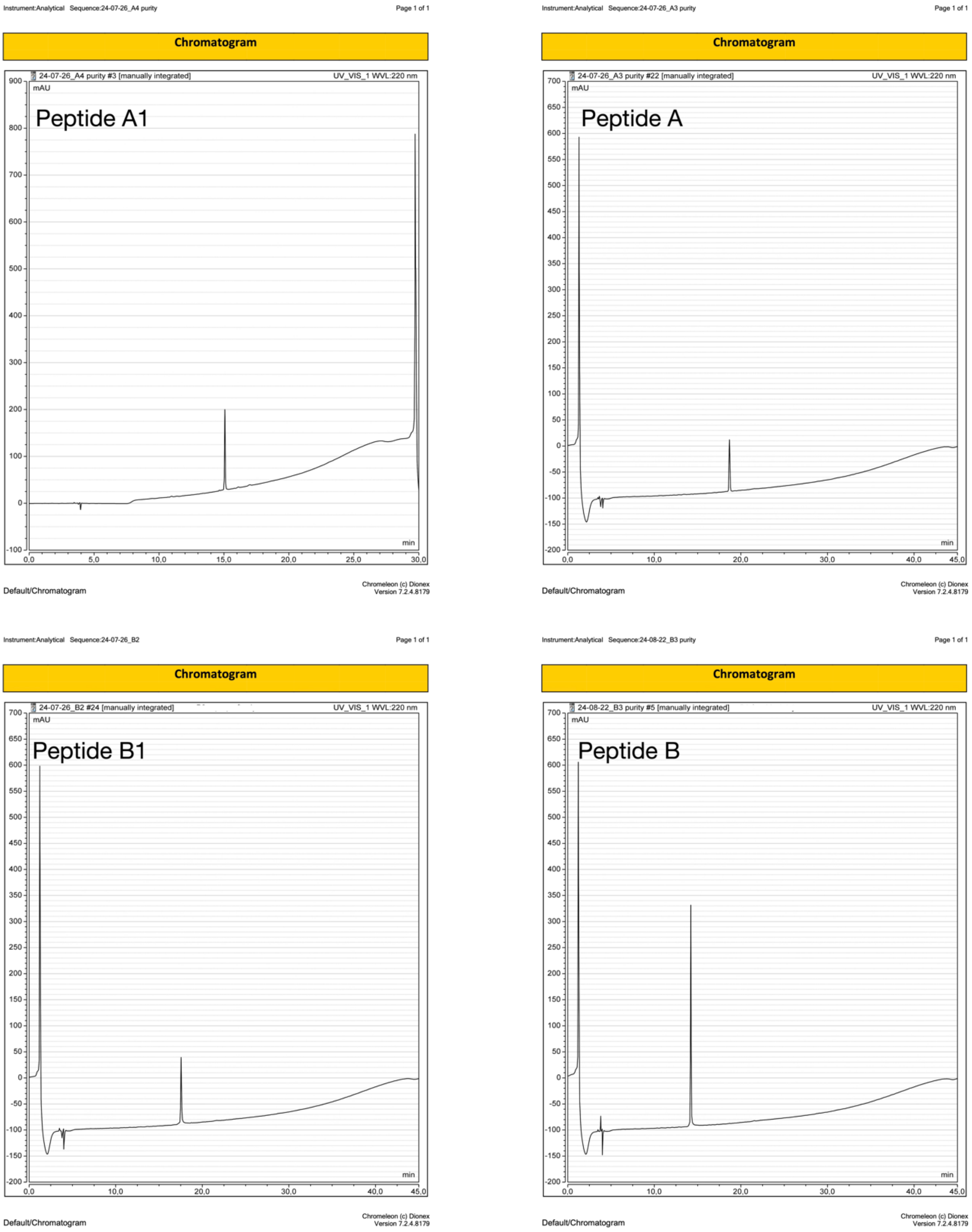

Supporting Figure 1. HPLC-chromatograms of the peptide (all of them are acetylated and amidated) fractions, used in this publication, to assess their purity after lyophilization (>98% for all peptides).

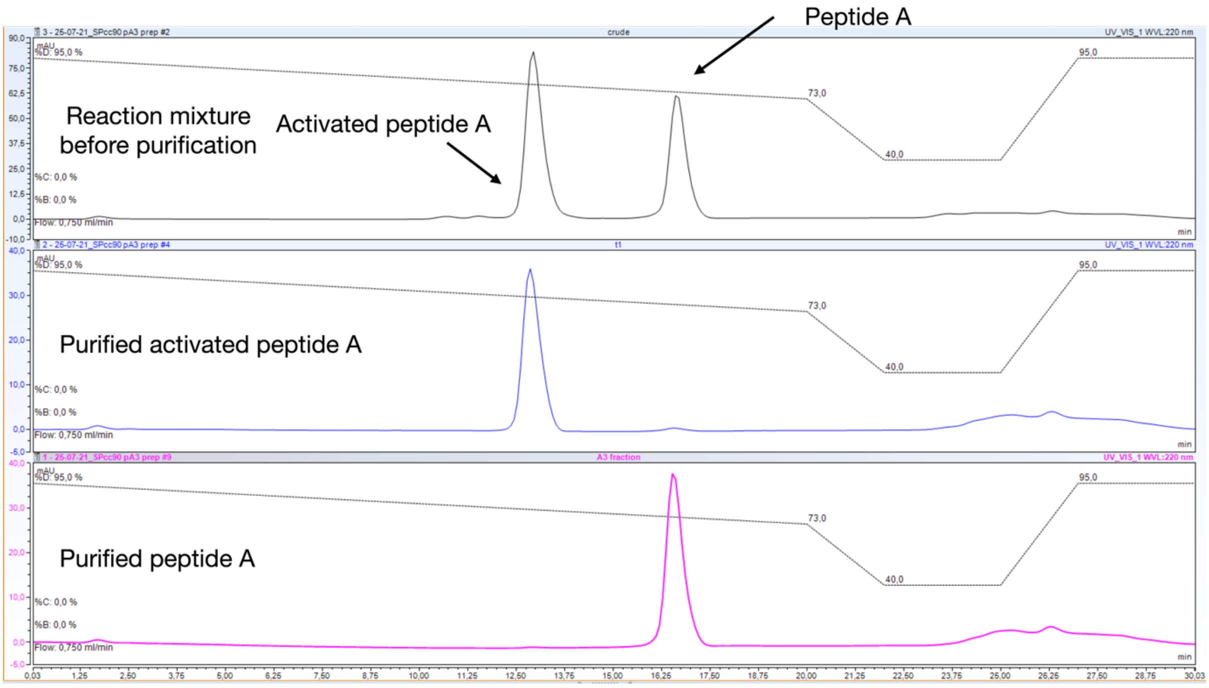

Supporting Figure 2. Characterization and purity assessment of the activated, phosphorylated peptide A before and directly after preparative HPLC.

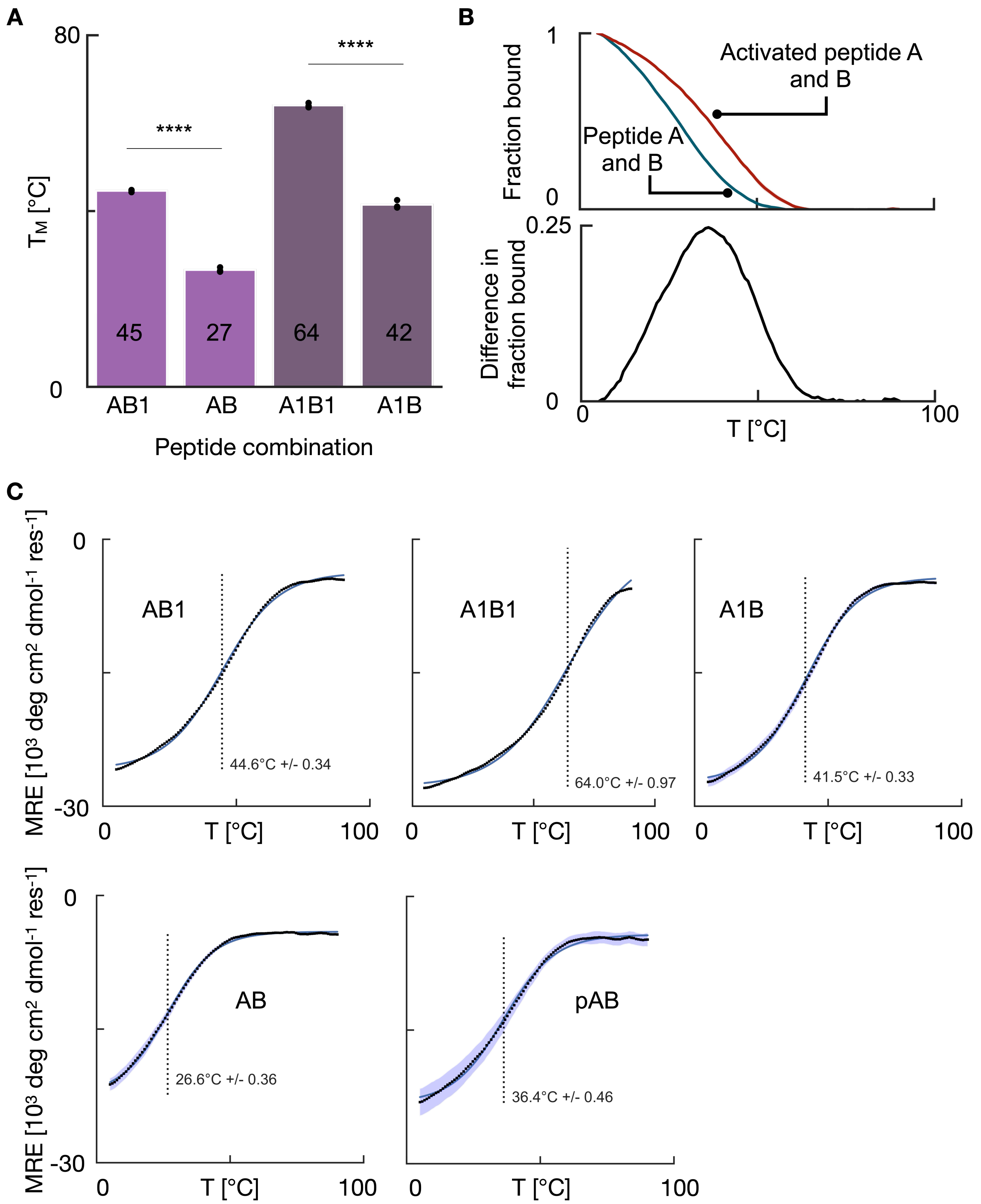

Supporting Figure 3. Melting Temperature determined with thermal melting by CD. A) Melting temperatures of different peptide combinations of 50 µM peptide A1/A and peptide B1/B in a 15 mM HEPES pH 6.5 buffered solution (light purple peptide A and dark purple peptide A1). (n = 3) **B)** The fraction bound in the coiled-coil state of peptide A (blue) or activated peptide A (red) and peptide B, derived from the melting curves with Eq. 1 (top), and the difference of deactivated and activated state

in fraction bound (bottom). (n = 3) **C)** Measured melting curves (black dots) and fits (blue lines) of of different peptide combinations of 50 µM peptide A1/A/pA and peptide B1/B in a 15 mM HEPES pH 6.5 buffered solution. (n = 3)

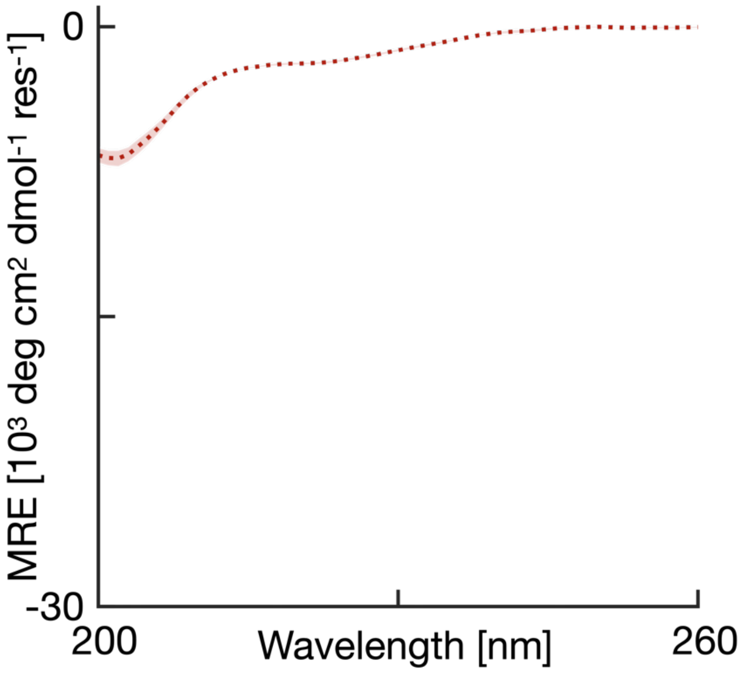

Supporting Figure 4. CD spectrum of activated peptide A 50 µM in 15 mM HEPES buffer at pH 6.5. (n = 3)

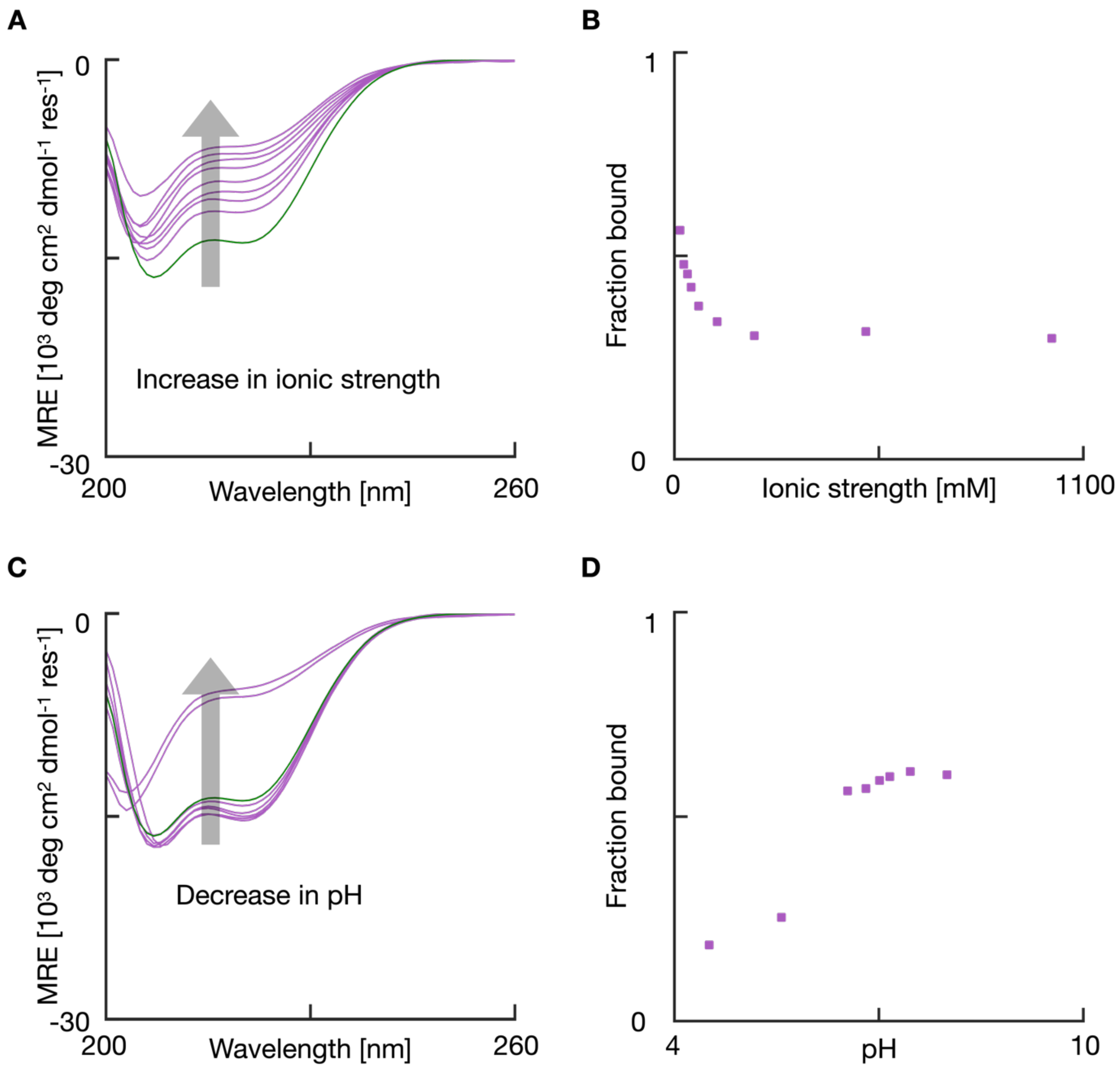

Supporting Figure 5. Effects of ionic strength and pH on the coiled-coil system. A) CD spectra of peptide A and B with increasing ionic strength leading to a decrease in helicity, and thus **B)** in the fraction bound. (n = 1) **C)** CD spectra of peptide A and B with decreasing pH, leading to a decrease in helicity and thus **D)** in the fraction bound. The green spectrum is the standard condition. (n = 1)

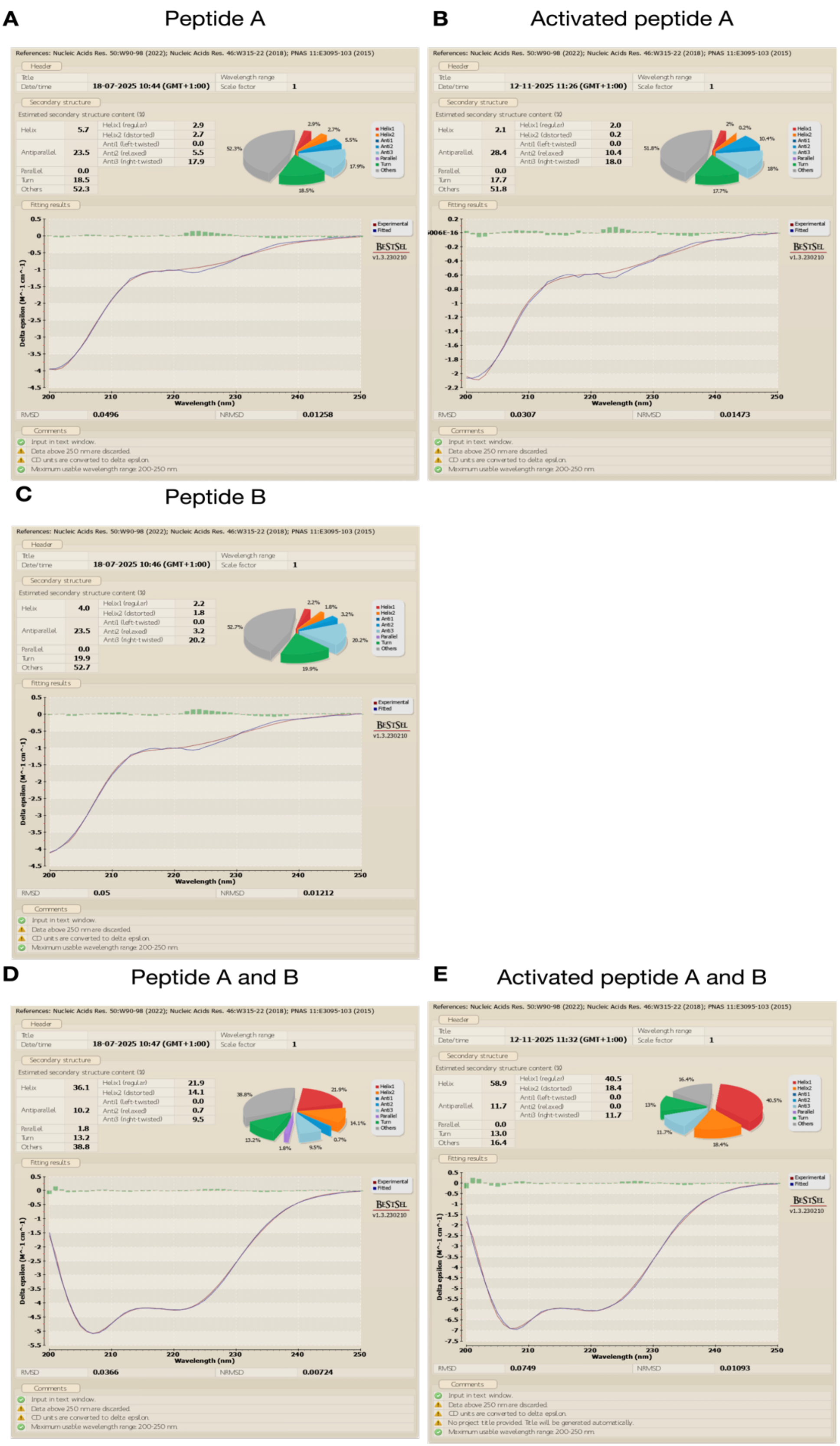

Supporting Figure 6. Estimated secondary structure content derived from the CD spectra (n = 3) of **A)** peptide A, **B)** peptide B, **C)** activated peptide A, **D)** peptide A and peptide B and **E)** activated peptide A and peptide B.[22]

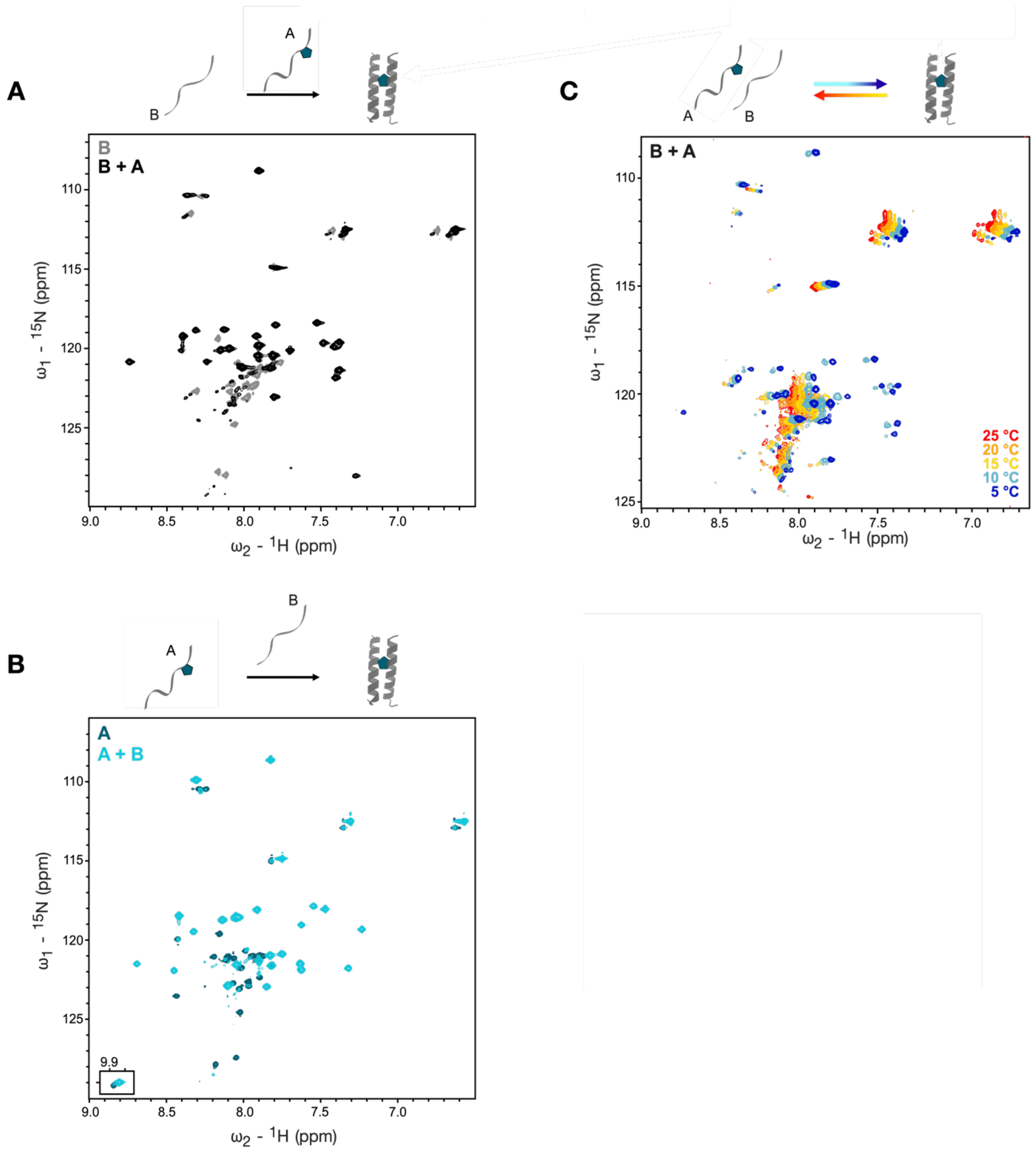

Supporting Figure 7. Characterization of the interaction between peptide A and peptide B by NMR. **A)** ^{1}H-^{15}N-HSQC spectrum of the ^{15}N-labeled peptide B in the presence of unlabeled peptide A, and **B)** ^{1}H-^{15}N-HSQC spectrum of the ^{15}N-labeled peptide A in the presence of unlabeled peptide B. Chemical shift changes and increased dispersion of amide proton chemical shifts indicate binding and an adaptation of ordered secondary structure. **C)** Temperature-dependent ^{1}H-^{15}N-HSQC of the peptide A and B coiled-coil system recorded from 5 to 25 °C. Exchange broadening of peaks at higher temperatures demonstrates the dynamic nature of the system.

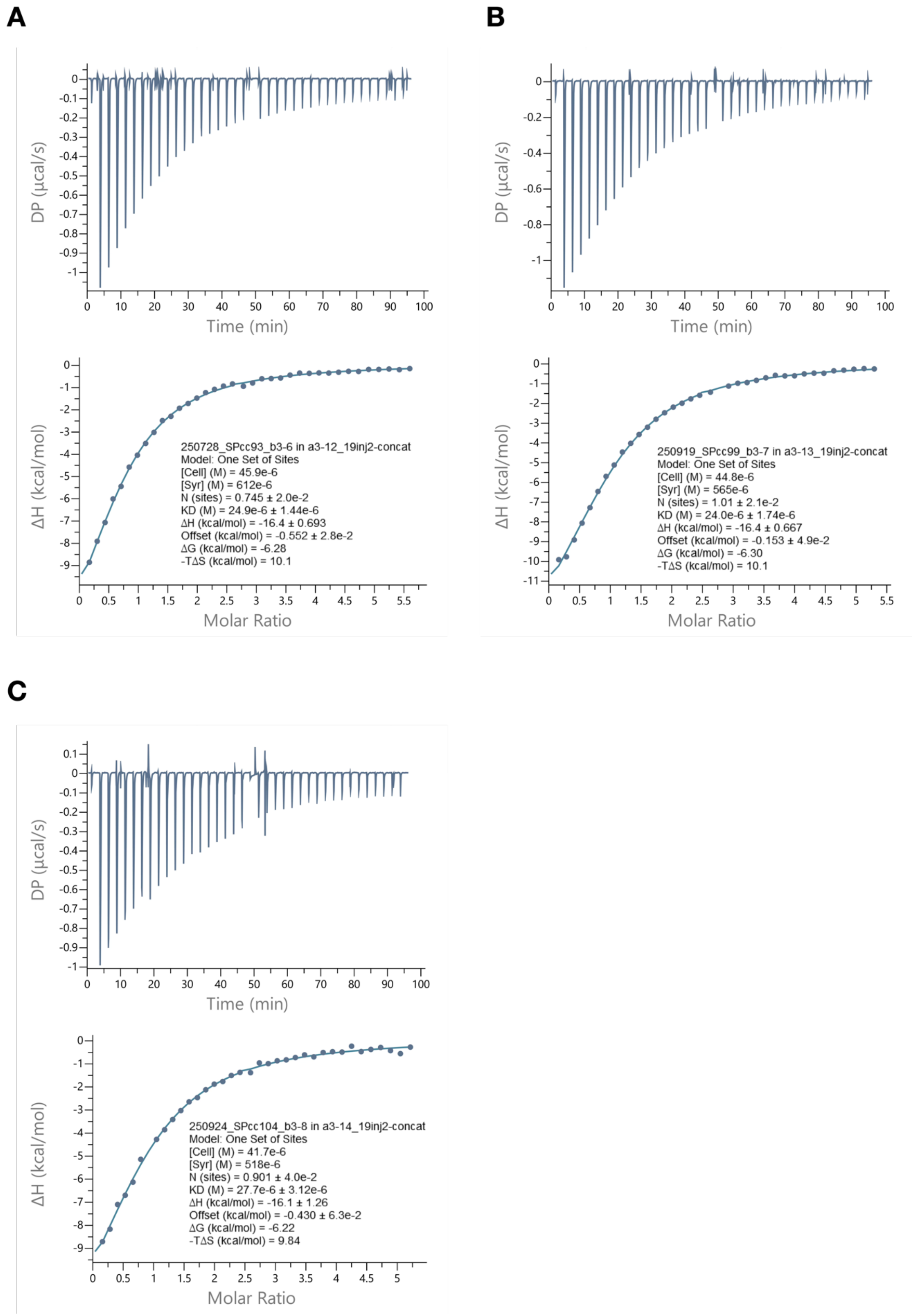

Supporting Figure 8. Replicates of the ITC titration of peptide A with peptide B. ITC titration curves given in differential power versus time and the enthalpy change versus molar ratio.

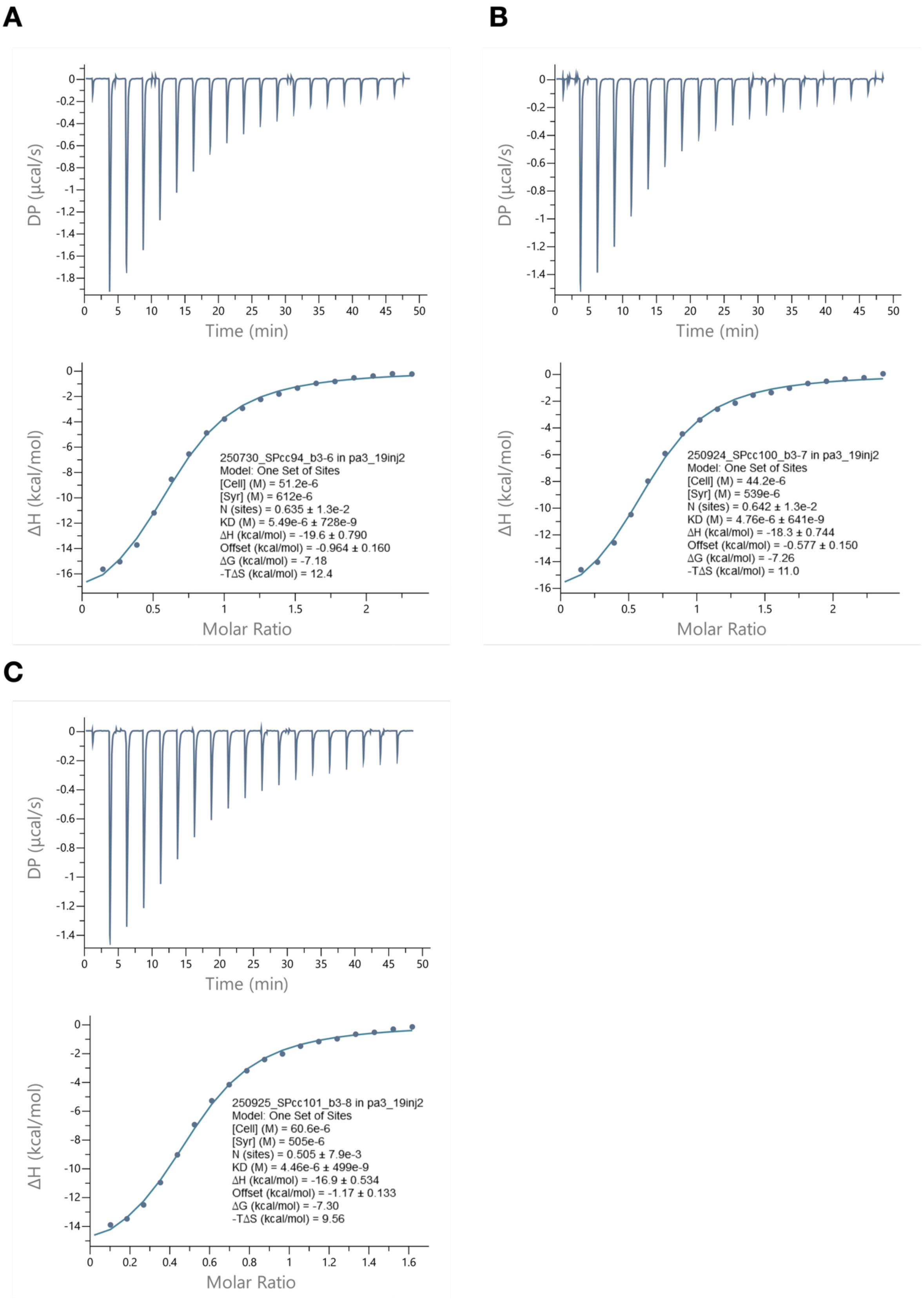

Supporting Figure 9. Replicates of the ITC titration of activated peptide A with peptide B. ITC titration curves given in differential power versus time and the enthalpy change versus molar ratio.

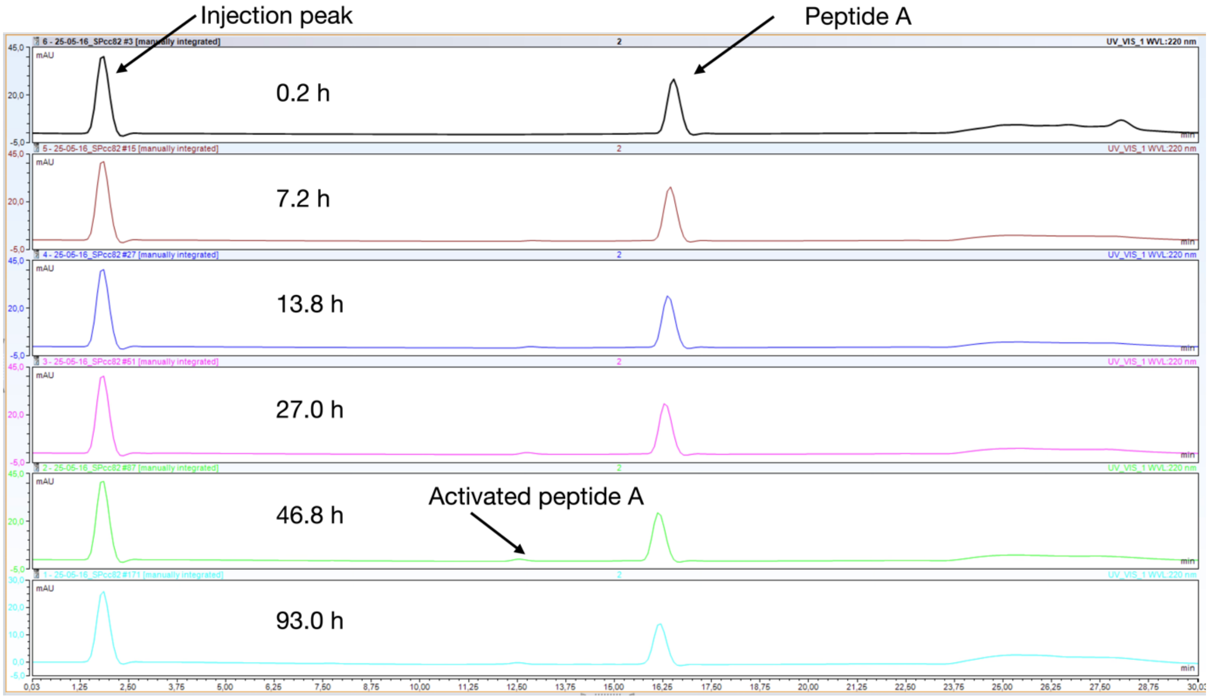

Supporting Figure 10. Representative time evolution of the signals in the analytical HPLC chromatogram of the activation of peptide A at the expense of MAP in the absence of peptide B measured at 220 nm.

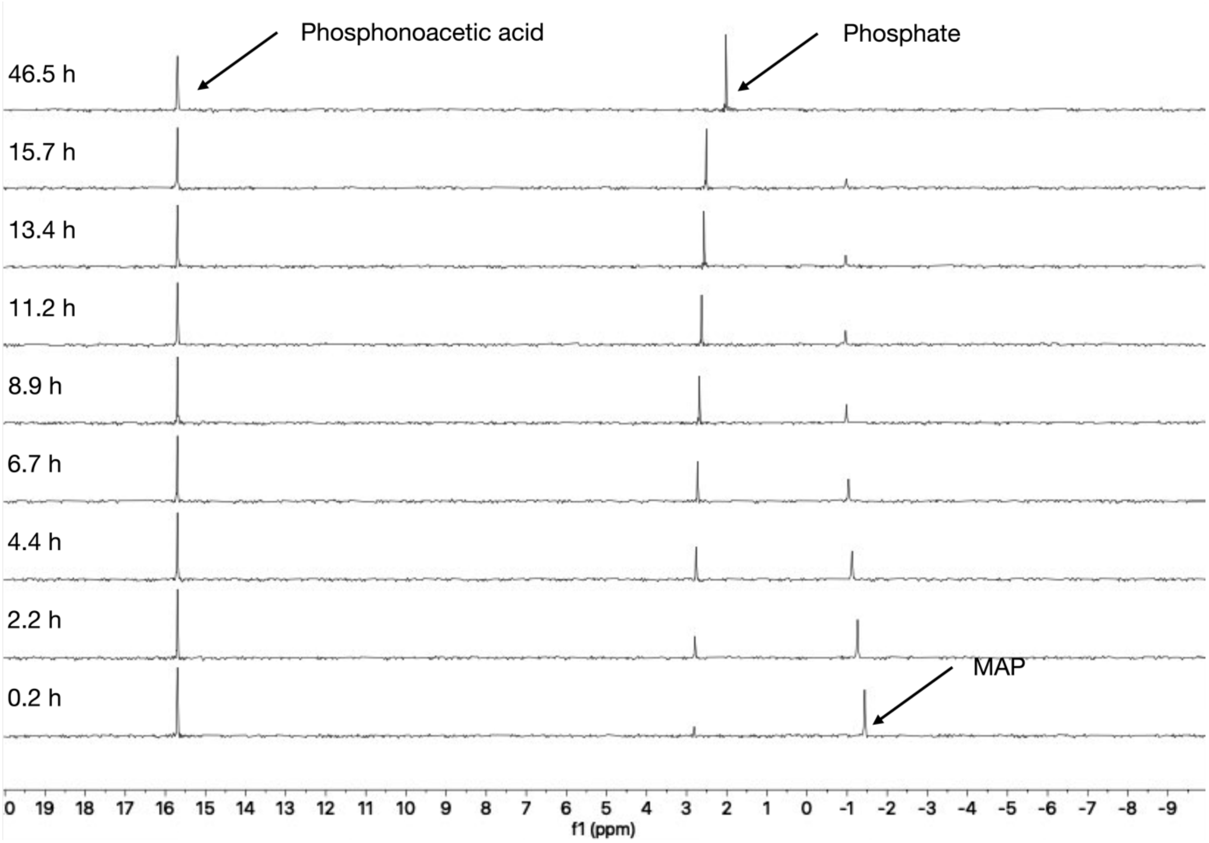

Supporting Figure 11. Representative time evolution of the chemical shifts in the ^{31}P-NMR spectra of the consumption of fuel in the presence of peptide A.

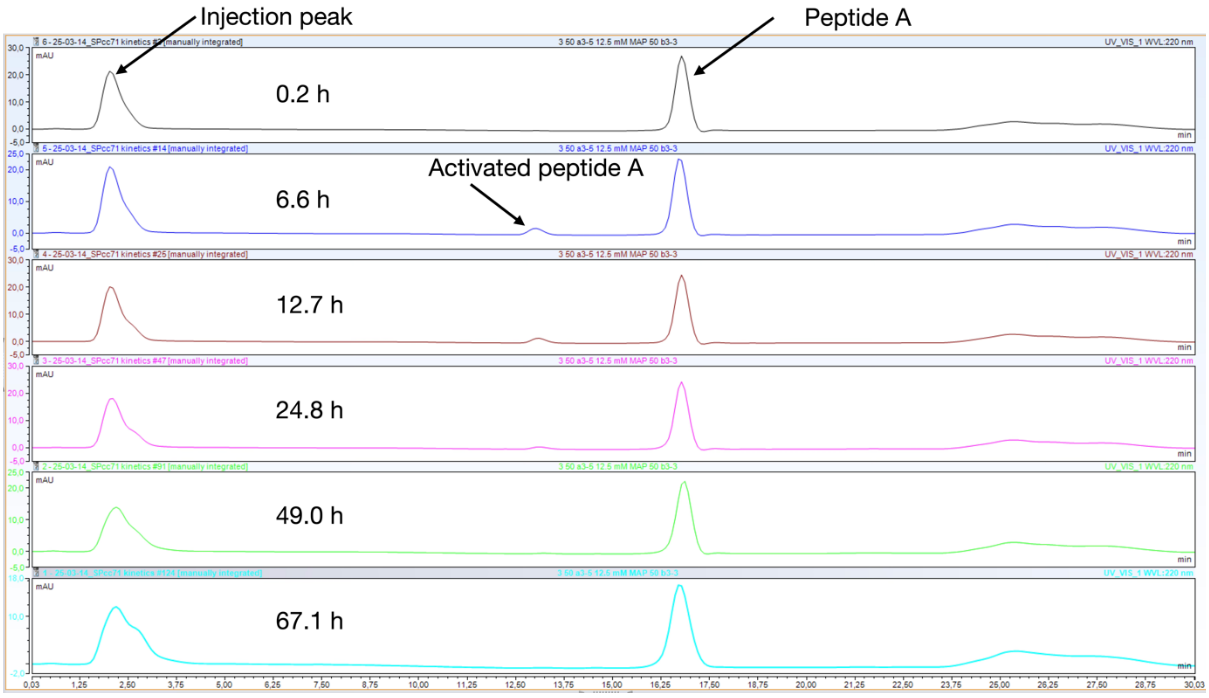

Supporting Figure 12. Representative time evolution of the signals in the analytical HPLC chromatogram of the activation of peptide A at the expense of MAP in the presence of peptide B measured at 220 nm.

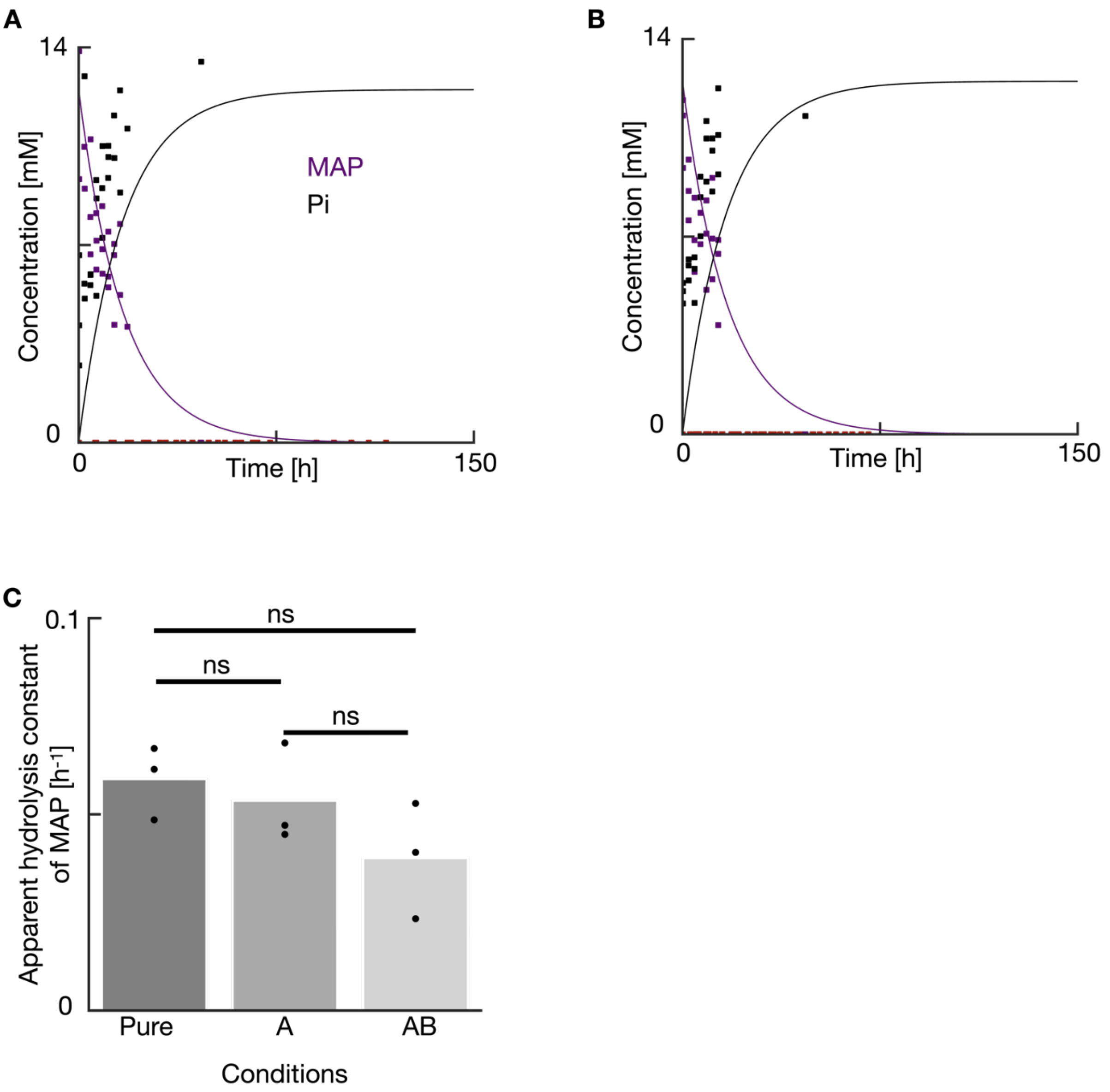

Supporting Figure 13. Kinetics of the coiled-coil system. Concentration profiles (squares) and kinetic profiles (lines) of the activation cycle of **A)** peptide A in the absence of peptide B and **B)** in the presence of peptide B, followed by ^{31}P- NMR and analytical HPLC. (n = 3) **E)** Bar plot of the (apparent) hydrolysis constants of MAP in the presence and absence of peptide A and/or peptide B. (n = 3)

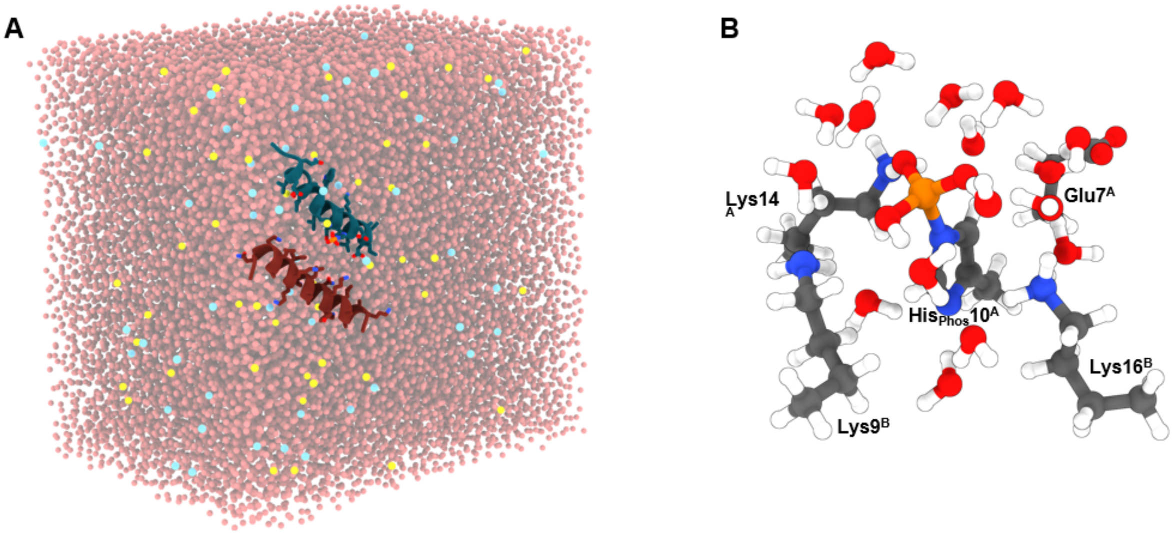

Supporting Figure 14. Multiscale simulation setups. A) MD simulation setup, showing the phosphorylated peptide dimer ($A_{Phos}B$) embedded in a TIP3P water box with 0.10 M NaCl. Peptides A and B are shown in teal and red, respectively. **B)** Quantum mechanical (QM) model of the parallel $A_{Phos}B$ system, extracted from the MD simulation. The model comprises the phosphorylated histidine ($His_{Phos}10^{A}$), interacting lysine residues ($Lys9^{B}$, $Lys14^{A}$, $Lys16^{B}$), and a nearby glutamate ($Glu7^{A}$), together with a few surrounding water molecules.

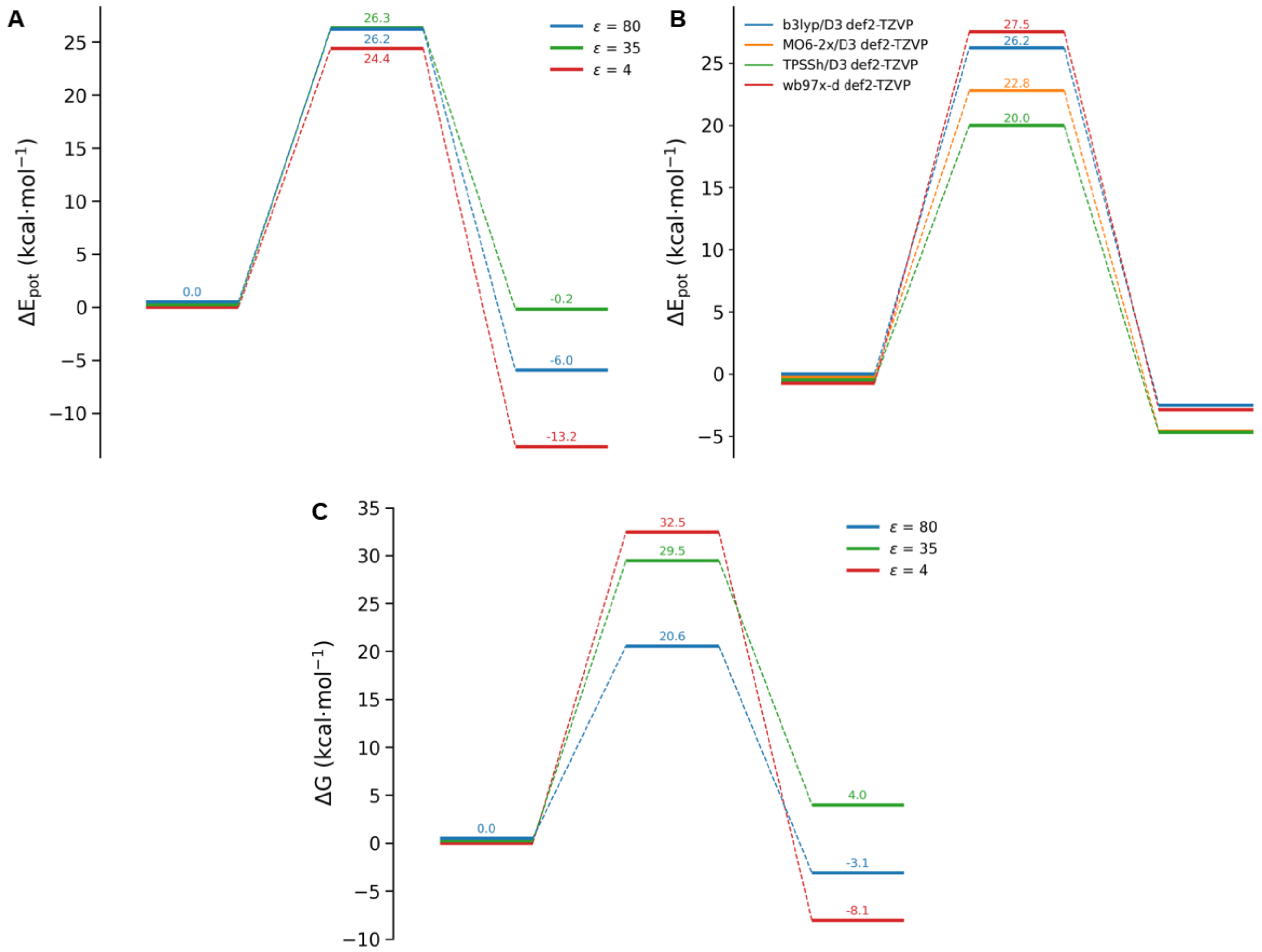

Supporting Figure 15. Benchmarking the DFT calculations. A) Potential energy profiles (ΔE_{pot}) at the B3LYP-D3/def2-TZVP level with varying dielectric constants (ε = 4, 35, 80) (see Table S2, systems Q1-Q3). **B)** Comparison of reaction barriers computed at different theory levels with ε=80 l (see Table S2: Q6-Q9). **C)** Free energy profiles (ΔG) at the B3LYP-D3/def2-TZVP level (see Table S4).

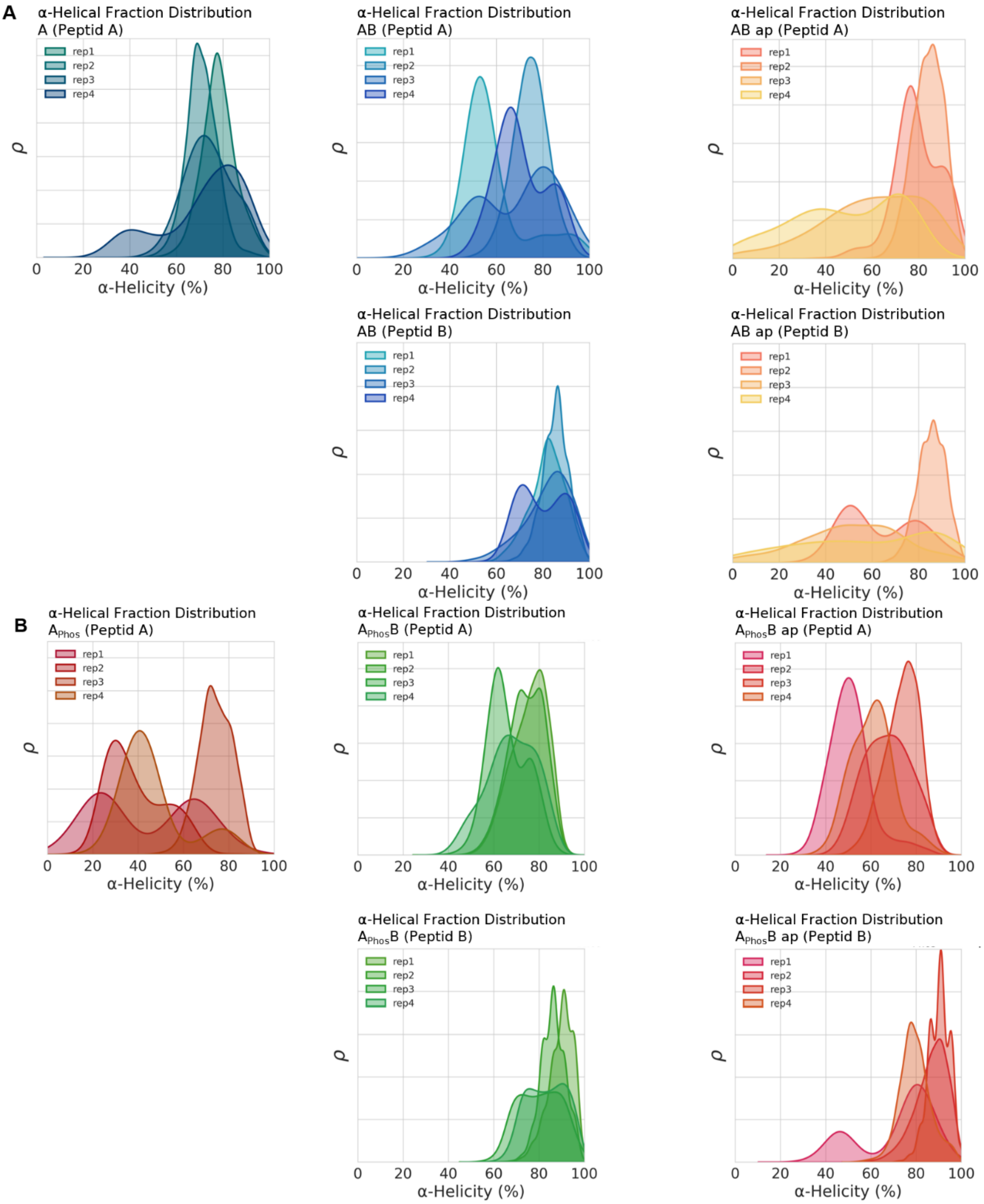

Supporting Figure 16. Distribution of α-helical fraction for the unphosphorylated and phosphorylated peptides. Density distributions of α-helicity for peptides A and B across four independent 500 ns MD replicas (see Table S1). **A)** Unphosphorylated systems show α-helical content for monomeric peptide A and dimeric AB configurations in both parallel and antiparallel orientations (Table S1: Simulations A, AB, AB ap). **B)** Corresponding phosphorylated systems (Table S1: Simulations A$_{Phos}$, A$_{Phos}$B, A$_{Phos}$B ap). The phosphorylation broadens the conformational ensemble of monomeric peptide A, while stabilizing the α-helical coiled-coil structure in the dimeric state.

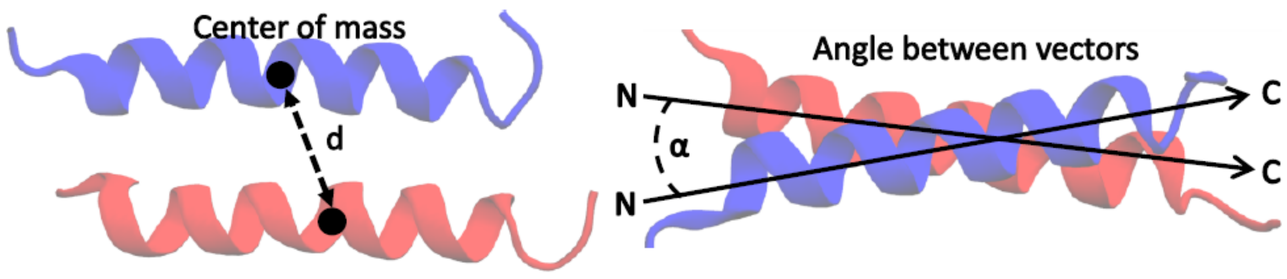

Supporting Figure 17. Schematic representation of geometric parameters used to monitor helix–helix interactions. Definition of center-of-mass (COM) distance (d) between the peptides and the interhelical angle (α), defined between vectors drawn from the N- to C-terminus along the helical segments of each peptide (residues 5–20). Low α values correspond to a parallel orientation of the helices, whereas high α values indicate an antiparallel arrangement.

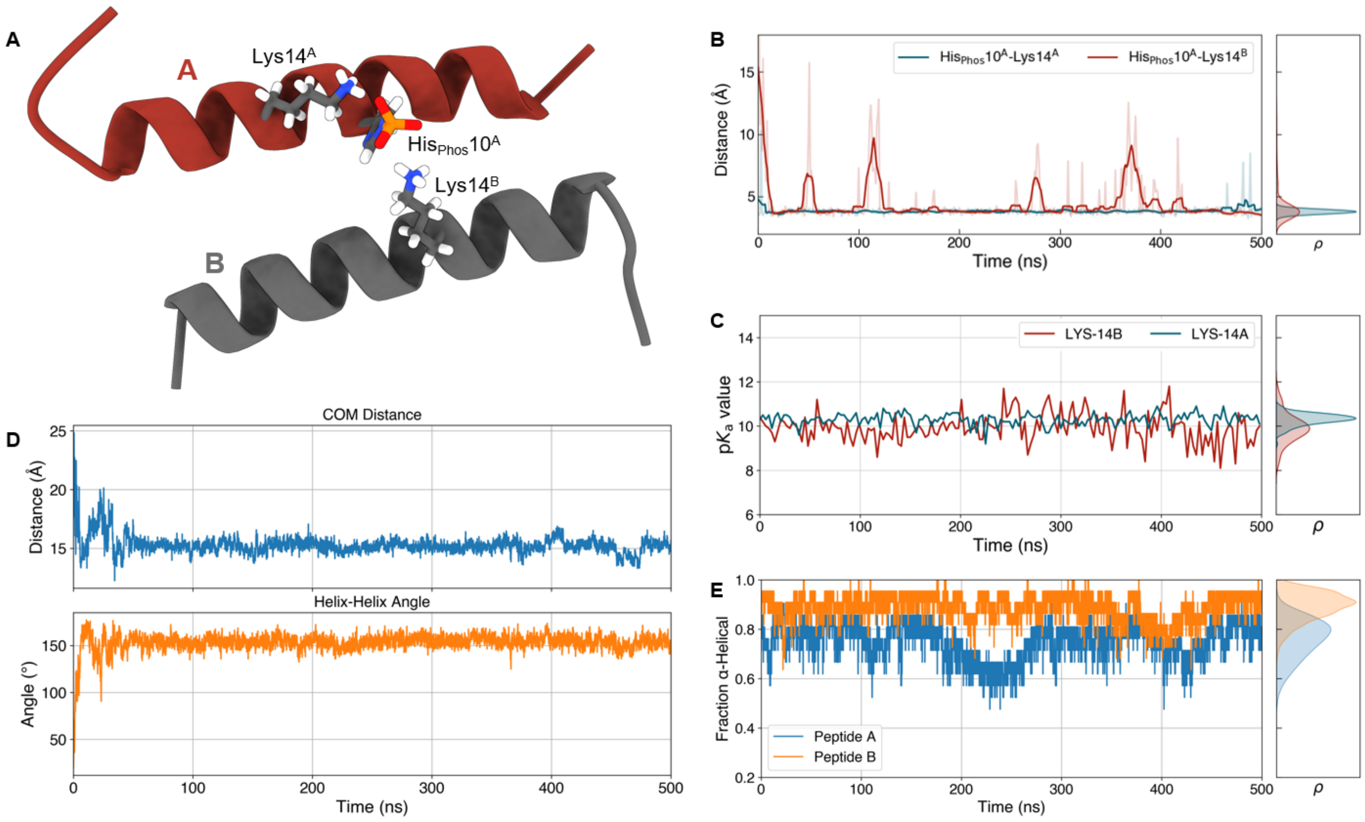

Supporting Figure 18. Dynamics and electrostatic analysis of the phosphorylated A_{Phos}B coiled-coil system of Simulation A_{Phos}B, replica 1. A) MD Snapshot 200 ns, showing the interaction between $His_{Phos}10^A$ and $Lys14^A$/ $Lys14^B$ within the coiled-coil interface (see Table S1). **B)** The $His_{Phos}10^A$–Lys distances and **C)** corresponding pK_a profiles, indicating transient pK_a lowering of the $Lys14^A$. **D)** Center-of-mass (COM) distance and helix–helix angle demonstrate a stable antiparallel association, while **E)** α-helical fractions show persistent secondary structure throughout the MD simulation.

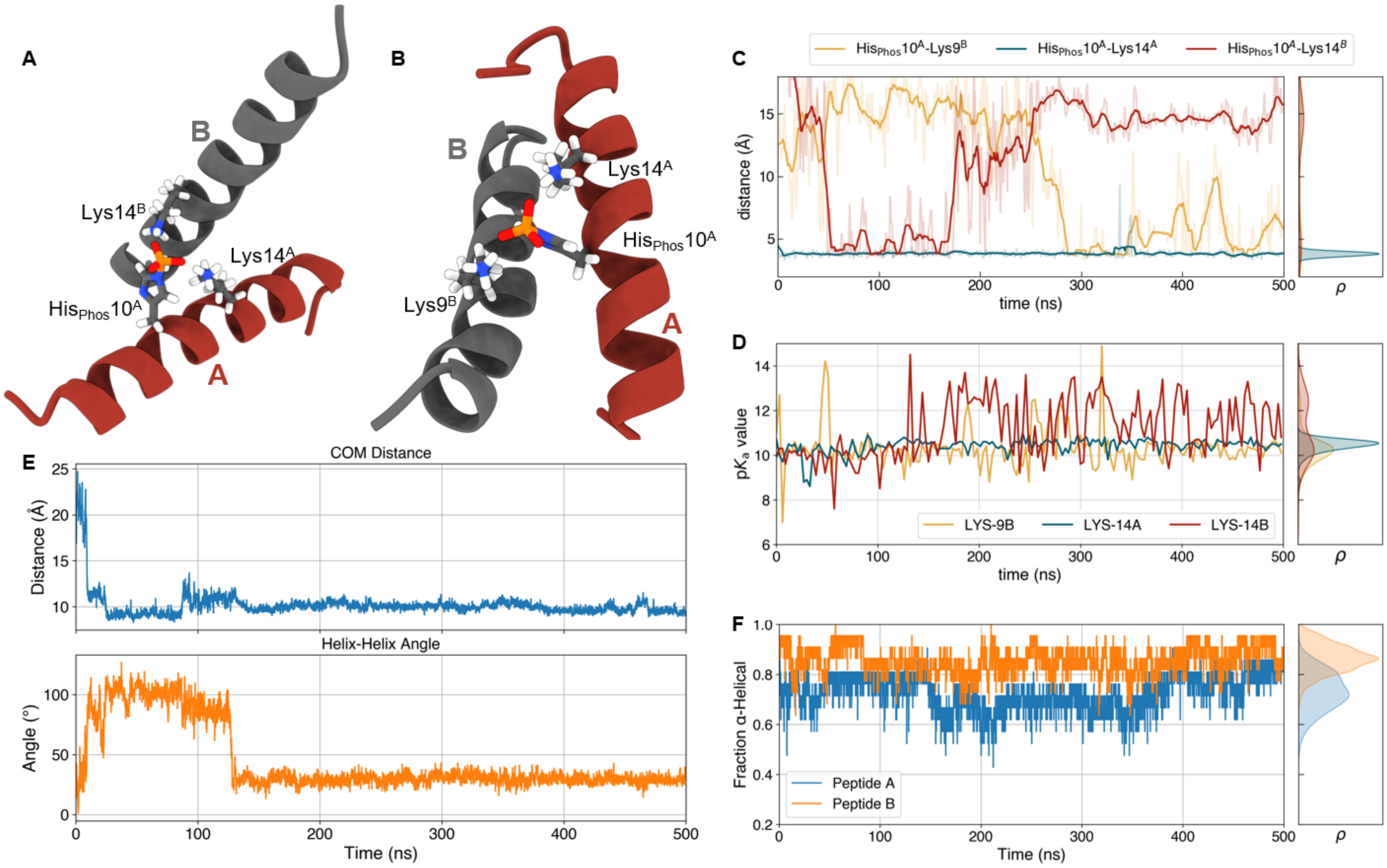

Supporting Figure 19. Dynamics and electrostatic analysis of the phosphorylated $A_{Phos}B$ coiled-coil system of Simulation $A_{Phos}B$, replica 2. (A, B) MD snapshots at 80 ns and 310 ns, showing conformational rearrangement of the $A_{Phos}B$ complex and the changing orientation of $His_{Phos}10^A$ relative to $Lys9^B$, / $Lys14^A$ and / $Lys14^B$ (see Table S1). **C)** The $His_{Phos}10^A$–Lys distances upon interaction. **D)** Corresponding pK_a profiles. **E)** Center-of-mass (COM) and helix–helix angle indicates a transition toward a more compact parallel arrangement after ~100 ns. (F) α-helicity content shows both helices remain predominantly α-helical throughout the MD simulations.

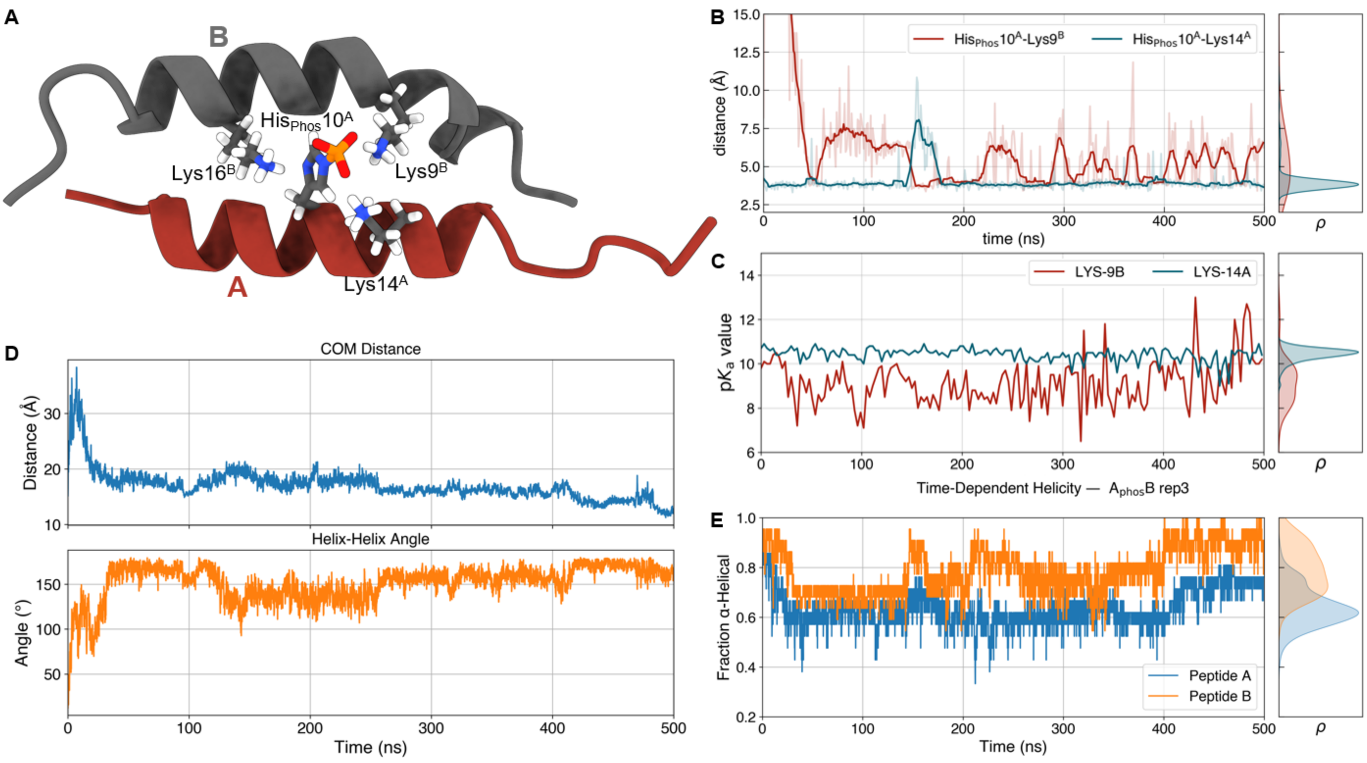

Supporting Figure 20. Dynamics and electrostatic analysis of the phosphorylated A_{Phos}B coiled-coil system Simulation A_{Phos}B, replica 3. A) MD snapshot at 205 ns showing the interaction between $His_{Phos}10^{A}$ and $Lys14^{A}$/ $Lys9^{B}$ (see Table S1). **B)** $His_{Phos}10^{A}$–Lys distances, and **C)** pK_a profiles, indicating transient pK_a lowering of $Lys9^{A}$ upon interaction. **D)** Center-of-mass (COM) distance and helix–helix angle demonstrates a stable association, whereas **E)** α-helical fractions show persistent secondary structure and even reformation of secondary structure throughout the MD simulations.

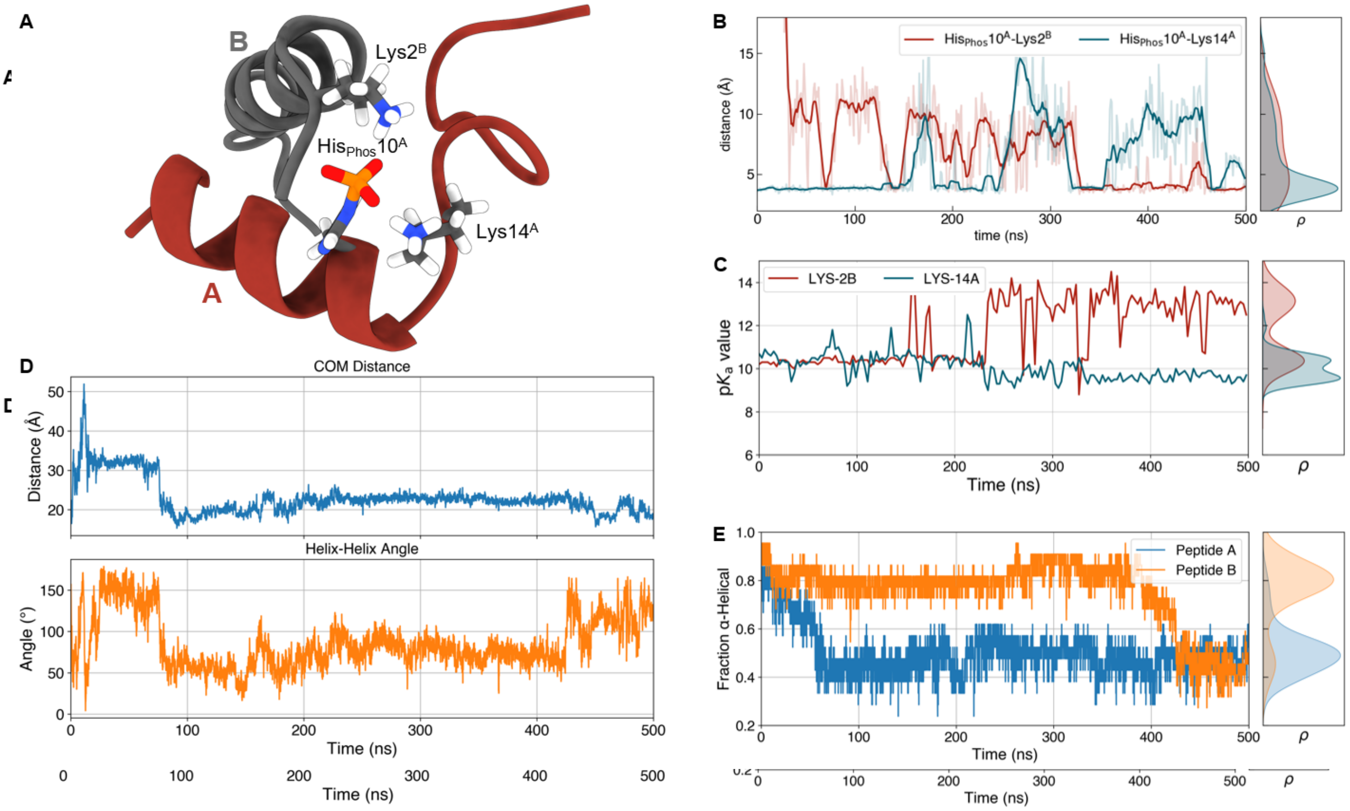

Supporting Figure 21. Dynamics and electrostatic analysis of the phosphorylated $A_{Phos}B$ coiled-coil system of Simulation $A_{Phos}B$, replica 4. A) MD snapshot at 306 ns and interaction between $His_{Phos}10^A$ and $Lys14^A$/ $Lys9^B$ within the coiled-coil interface (see Table S1). **B)** The $His_{Phos}10^A$–Lys distances. **C)** Corresponding pK_a profiles, indicating transient pK_a lowering of $Lys9^A$ upon interaction. **D)** Center-of-mass (COM) distance and helix–helix angle demonstrate a stable association. **E)** α-helical fractions show persistent secondary structure throughout the MD simulation.

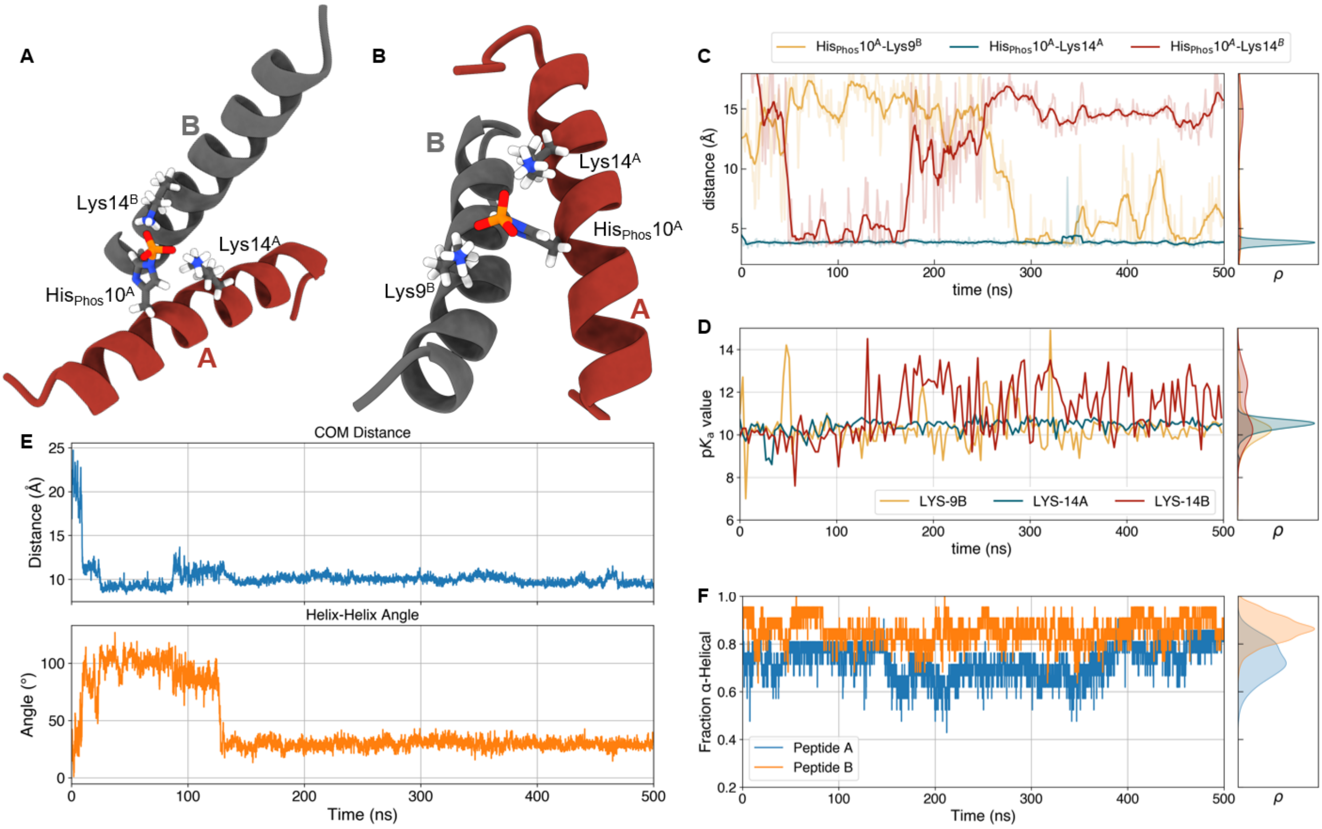

Supporting Figure 22. Dynamics and electrostatic analysis of the phosphorylated A_{Phos}B antiparallel coiled-coil system of simulation A_{Phos}B ap, replica 1. A) MD snapshot at 323 ns and interaction between $His_{Phos}10^A$ and $Lys14^A$/ $Lys2^B$ (see Table S1). **B)** The $His_{Phos}10^A$–Lys distances, indicating a dynamic formation and dissociation of the contents. **C)** Corresponding pK_a profiles, with a pK_a shift of $Lys2^B$ due to salt-bridge formation with a nearby glutamate. **D)** Center-of-mass (COM) distance and helix–helix angle during association of the peptides. **E)** α-helical fractions show disruption of helicity with the formation of the salt bridge of $Lys2^B$.

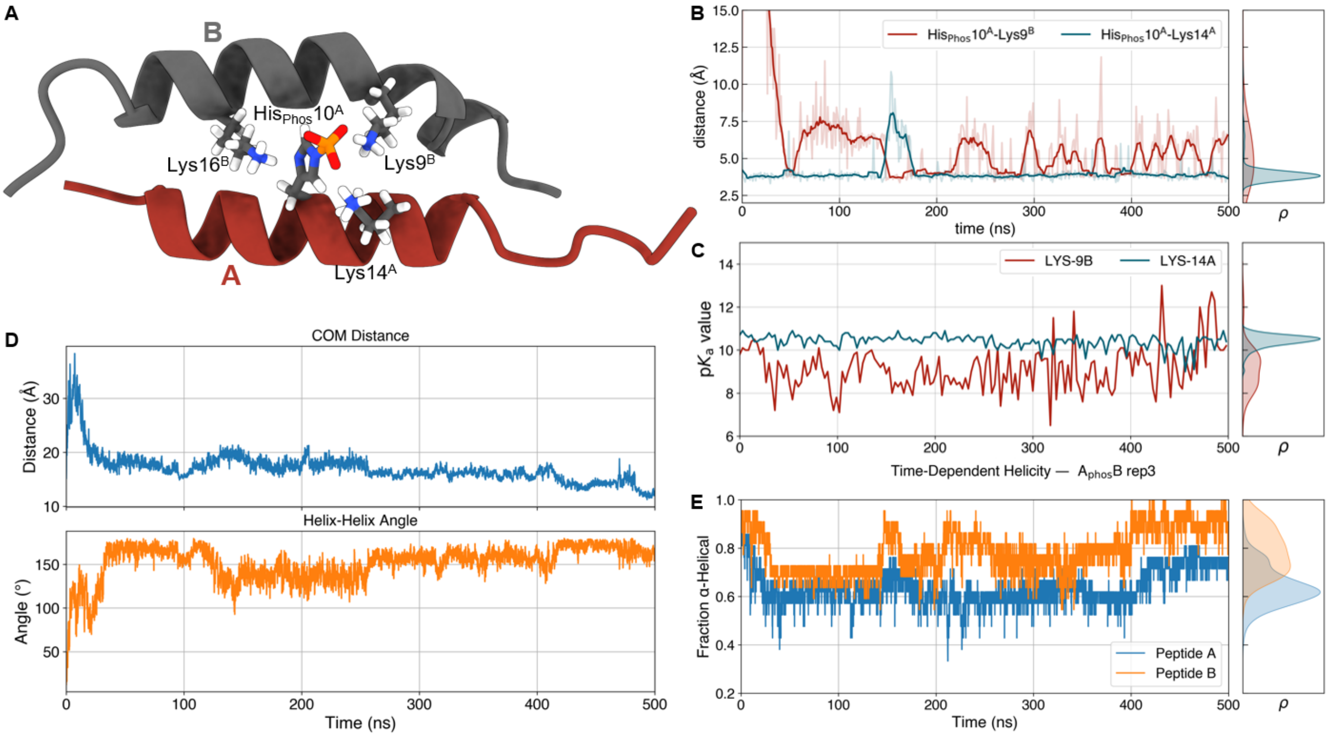

Supporting Figure 23. Dynamics and electrostatic analysis of the phosphorylated $A_{Phos}B$ antiparallel coiled-coil system simulation $A_{Phos}B$ ap, replica 2. (A, B) MD snapshots at 96 ns and 275 ns, showing conformational rearrangement of the $A_{Phos}B$ complex and $His_{Phos}10^{A}$- $Lys14^{B}$- $Lys14^{A}$-$Lys21^{B}$ interactions (see Table S1). **C)** The $His_{Phos}10^{A}$–Lys distances reveals alternating interaction patterns over the simulation. **D)** Corresponding pK_a profiles. **E)** Center-of-mass (COM) distance and helix–helix angle indicates a transition toward a more compact parallel arrangement after ~100 ns. (F) α-helical content, indicating that both helices remain predominantly α-helical throughout the MD simulations.

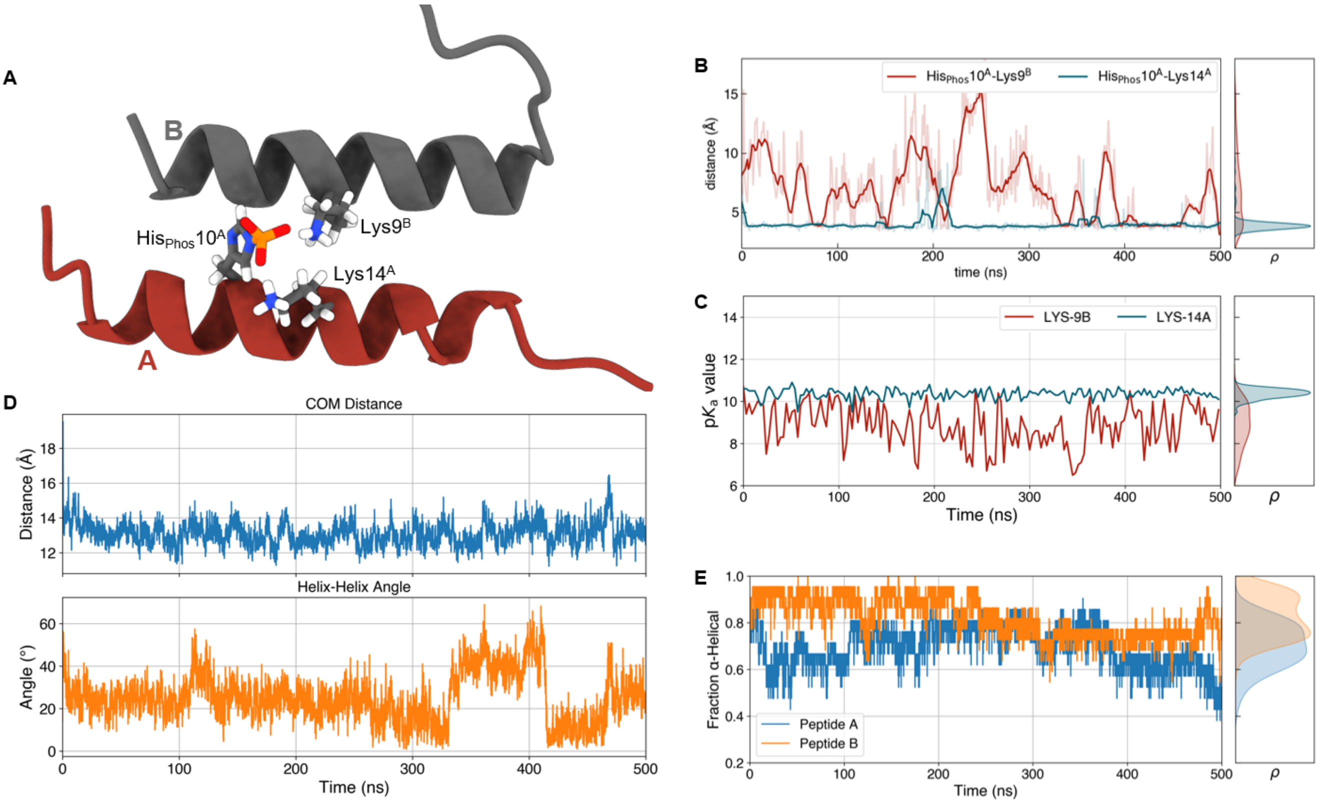

Supporting Figure 24. Dynamics and electrostatic analysis of the phosphorylated A$_{\text{Phos}}$B antiparallel coiled-coil system Simulation A$_{\text{Phos}}$B ap, replica 3. A) MD snapshot at 230 ns, showing conformational rearrangement of the A$_{\text{Phos}}$B complex and the His$_{\text{Phos}}$10^{A}-Lys14^{B}, Lys14^{A} interaction (see Table S1). **B)** The His$_{\text{Phos}}$10^{A}–Lys distances and interactions during the simulations. **C)** Corresponding pK_a profiles, showing significant pK_a shifts upon coiled-coil formation. **D)** Center-of-mass (COM) and helix–helix angle indicates a compact arrangement. **E)** α-helical content, indicates that both helices remain predominantly α-helical throughout the MD simulations.

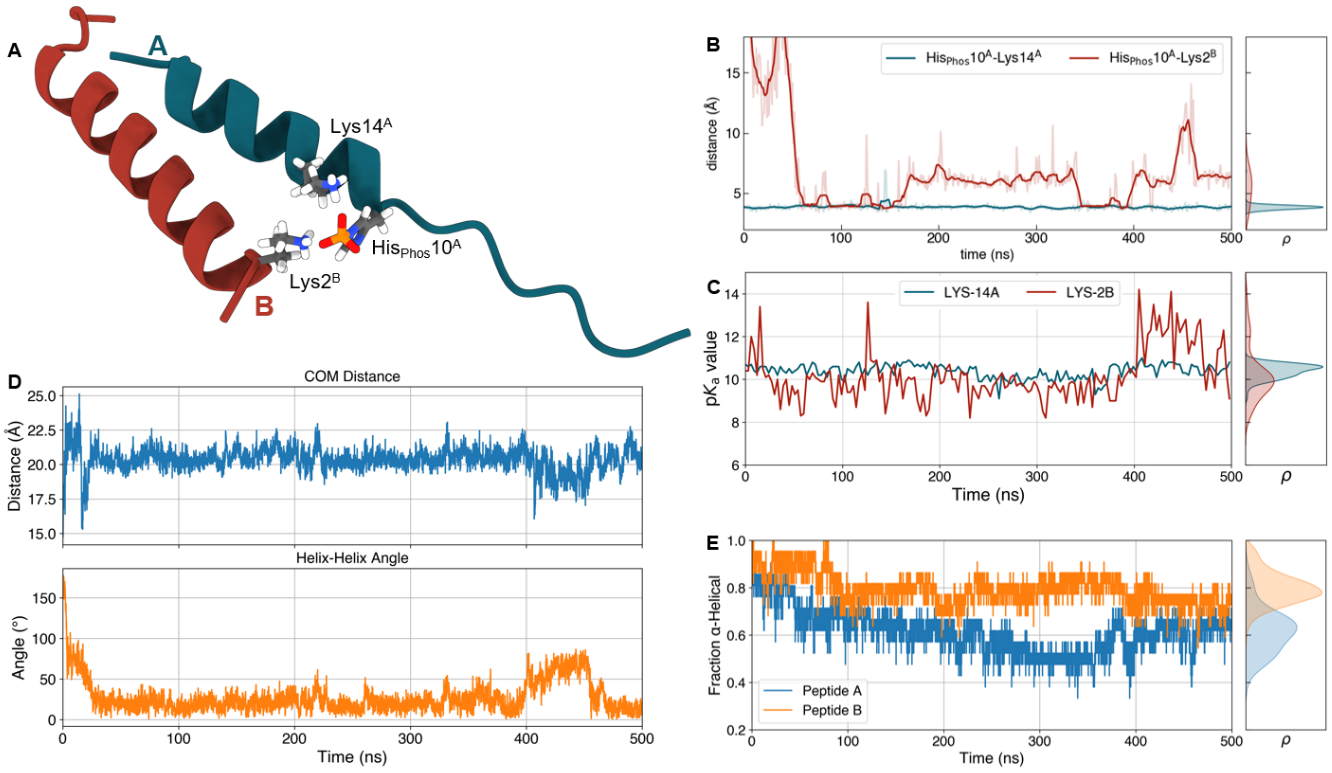

Supporting Figure 25. Dynamics and electrostatic analysis of the phosphorylated $A_{Phos}B$ antiparallel coiled-coil system Simulation $A_{Phos}B$ ap, replica 4. A) MD snapshot at 360 ns, showing conformational rearrangement of the $A_{Phos}B$ complex and shift of the $His_{Phos}10^A$-$Lys2^B$- $Lys14^A$ interaction (see Table S1). **B)** The $His_{Phos}10^A$–Lys distances and interaction patterns over the MD simulation. **D)** Corresponding pK_a profiles, showing a pK_a shift of $Lys2^B$. **D)** Center-of-mass (COM) distance and helix–helix angle over time. **E)** α-helical content during MD simulations, indicating that both helices remain predominantly α-helical, with a tendency of unfolding of peptide A.

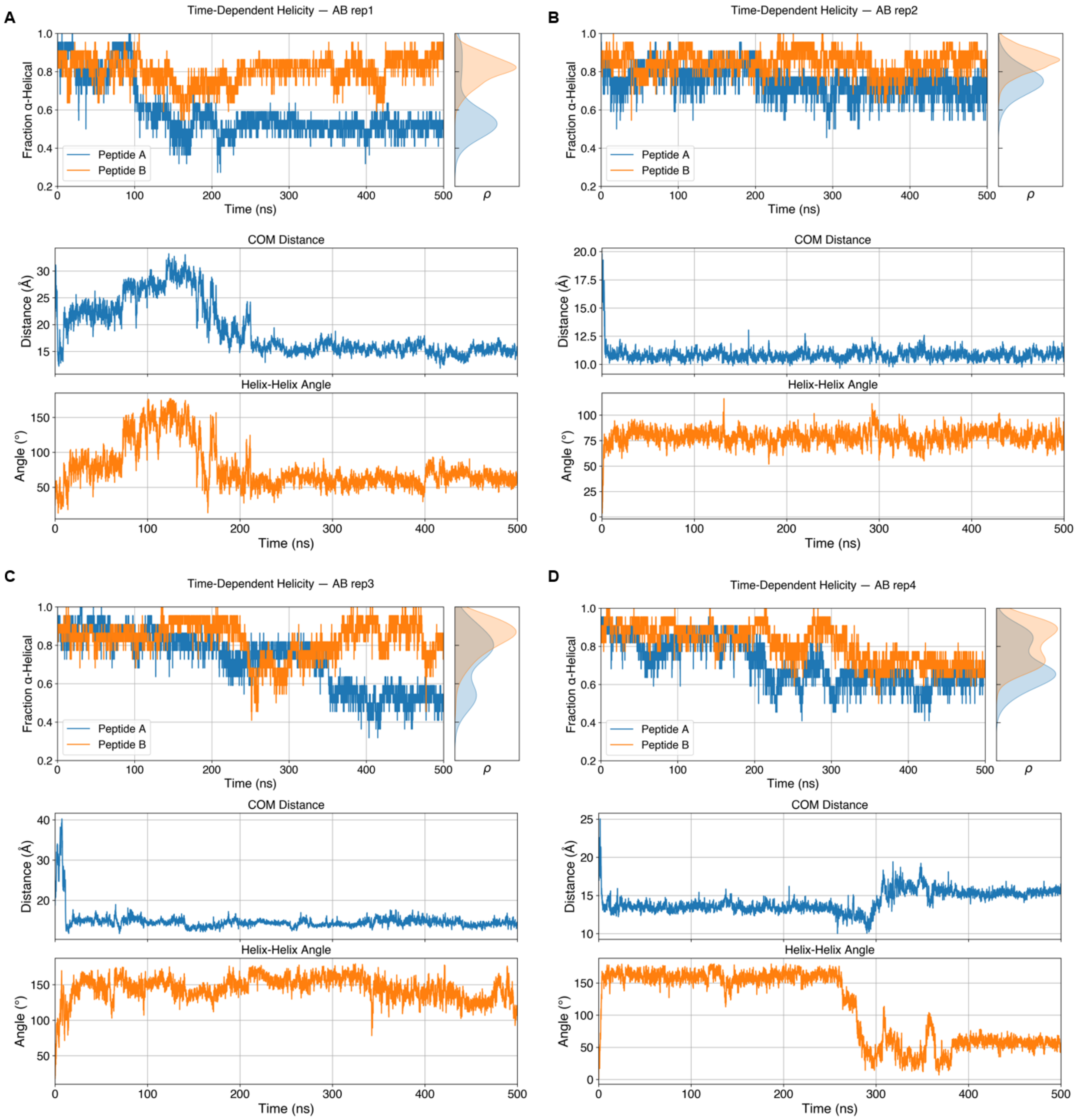

Supporting Figure 26. Dynamics of the unphosphorylated AB coiled-coil system. (A-D) Dynamics of α-helical content, helix–helix center-of-mass (COM) distance, and interhelical angle for replicas 1 – 4 (Table S1: Simulation AB**)**. Both simulations show moderate α-helical stability for peptides A and B, with transient unfolding and refolding events over the 500 ns MD simulations. The COM distance remains stable, while the helix–helix angle varies, indicating association and dissociation events for the weakly interacting antiparallel dimer. D) Re-orientation of the peptide from parallel to antiparallel conformation.

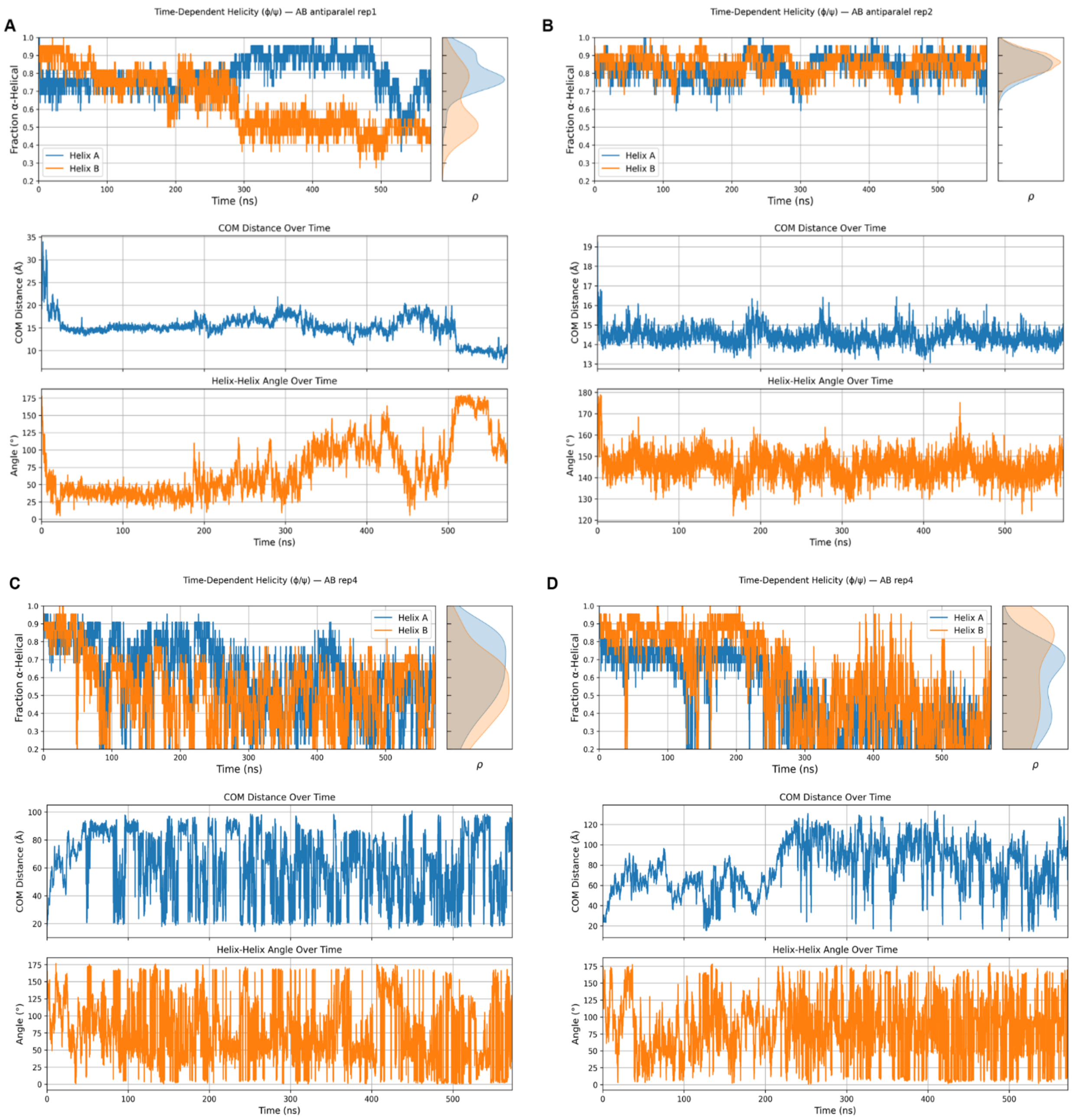

Supporting Figure 27. Dynamics of the unphosphorylated antiparallel AB peptide (AB-ap) system in the coiled-coil state. (A–D) Dynamics and α-helical content, helix–helix center-of-mass (COM) distance, and interhelical angle for the four independent replicas of the non-phosphorylated in antiparallel starting configuration (Table S1: Simulation AB ap**)**. In A and B, both helices remain partially α-helical with transient unfolding events. In C) and D), no stable interhelical interactions are established, and both peptides dissociate.

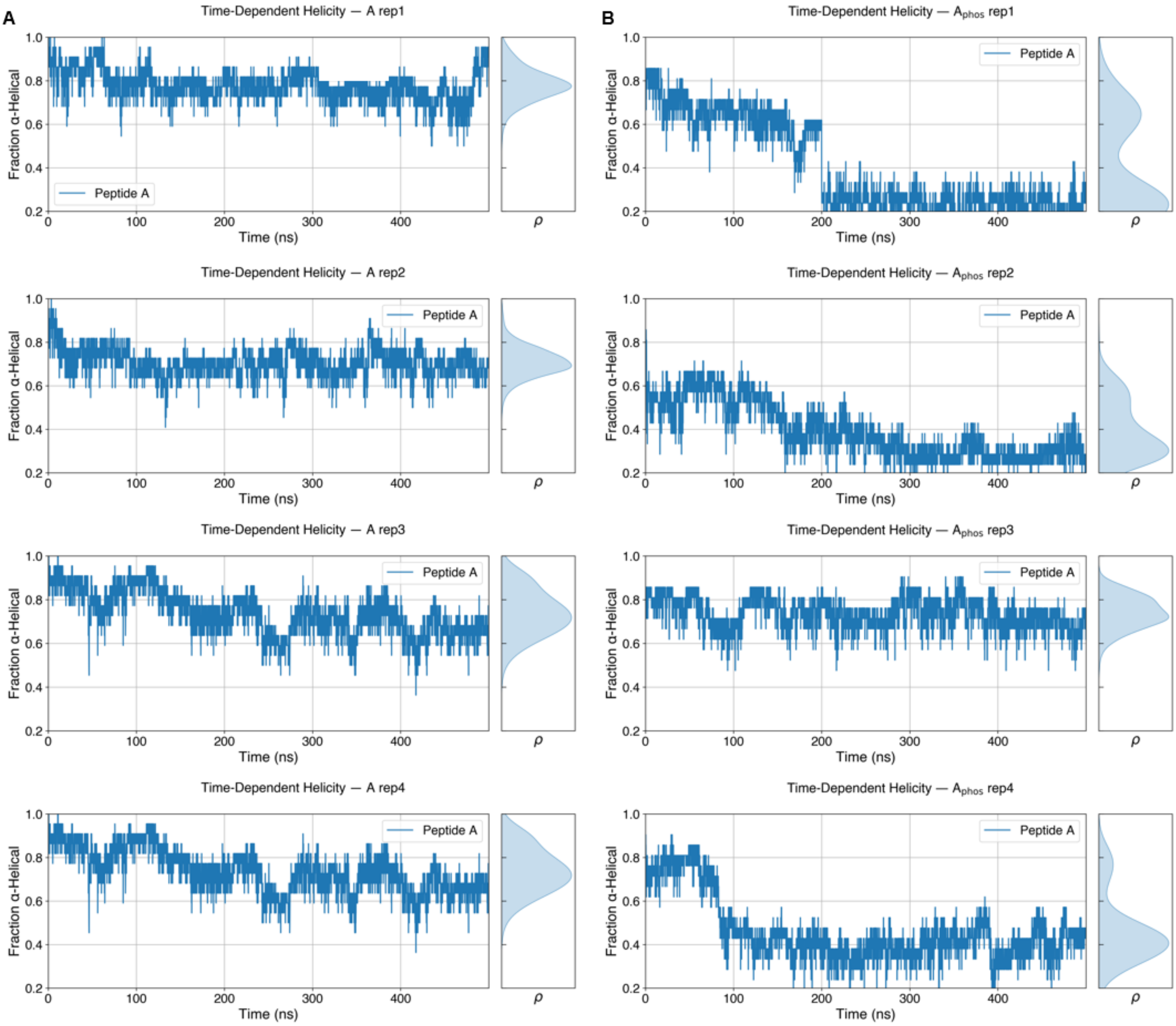

Supporting Figure 28. Dynamics of peptide A in the unphosphorylated and phosphorylated states. A) Unphosphorylated peptide A with the His10 in the N_{δ} protonated state (Table S1: Simulation A), and **B)** Phosphorylated peptide A (His_{10}–P) (Table S1: Simulation A_{Phos}), showing the fraction of α-helical residues from the MD simulations with the right panels showing the corresponding probability density distributions (ρ) of α-helicity. Phosphorylation of His10 results in an increased fluctuation of the α-helical content, and destabilization of the helical structure.

References

[1] Y.-F. Wei, H. R. Matthews, *Methods in enzymology* **1991**, *200*, 388-414.
[2] T. J. Henderson, *Analytical Chemistry* **2002**, *74*, 191-198.
[3] R. Rigger, A. Rück, C. Hellriegel, R. Sauermoser, F. Morf, K. Breitruck, M. Obkircher, *Journal of AOAC International* **2017**, *100*, 1365-1375.
[4] J. Bonet, Z. Harteveld, F. Sesterhenn, A. Scheck, B. E. Correia, *BMC bioinformatics* **2019**, *20*, 240.
[5] L. Harrington, J. M. Fletcher, T. Heermann, D. N. Woolfson, P. Schwille, *Nature communications* **2021**, *12*, 1-11.
[6] W. Lee, M. Tonelli, J. L. Markley, *Bioinformatics* **2015**, *31*, 1325-1327.
[7] J. Abramson, J. Adler, J. Dunger, R. Evans, T. Green, A. Pritzel, O. Ronneberger, L. Willmore, A. J. Ballard, J. Bambrick, *Nature* **2024**, *630*, 493-500.
[8] W. L. Jorgensen, J. Chandrasekhar, J. D. Madura, R. W. Impey, M. L. Klein, *The Journal of chemical physics* **1983**, *79*, 926-935.
[9] J. B. Klauda, R. M. Venable, J. A. Freites, J. W. O'Connor, D. J. Tobias, C. Mondragon-Ramirez, I. Vorobyov, A. D. MacKerell Jr, R. W. Pastor, *The journal of physical chemistry B* **2010**, *114*, 7830-7843.
[10] J. C. Phillips, R. Braun, W. Wang, J. Gumbart, E. Tajkhorshid, E. Villa, C. Chipot, R. D. Skeel, L. Kale, K. Schulten, *Journal of computational chemistry* **2005**, *26*, 1781-1802.
[11] W. Humphrey, A. Dalke, K. Schulten, *Journal of molecular graphics* **1996**, *14*, 33-38.
[12] N. A. Baker, D. Sept, S. Joseph, M. J. Holst, J. A. McCammon, *Proceedings of the National Academy of Sciences* **2001**, *98*, 10037-10041.
[13] T. Meyer, E.-W. Knapp, *Journal of chemical theory and computation* **2015**, *11*, 2827-2840.
[14] P. Hohenberg, W. Kohn, *Physical review* **1964**, *136*, B864.
[15] S. Grimme, J. Antony, S. Ehrlich, H. Krieg, *The Journal of chemical physics* **2010**, *132*.
[16] A. D. Becke, *The Journal of chemical physics* **1993**, *98*, 5648-5652.
[17] C. Lee, W. Yang, R. G. Parr, *Physical review B* **1988**, *37*, 785.
[18] A. Schaefer, H. Horn, R. Ahlrichs, *J. chem. Phys* **1992**, *97*, 2571.
[19] F. Furche, R. Ahlrichs, C. Hättig, W. Klopper, M. Sierka, F. Weigend, *Wiley Interdisciplinary Reviews: Computational Molecular Science* **2014**, *4*, 91-100.
[20] S. M. Poprawa, M. Stasi, B. A. Kriebisch, M. Wenisch, J. Sastre, J. Boekhoven, *Nature Communications* **2024**, *15*, 4204.
[21] G. Ragazzon, L. J. Prins, *Nature nanotechnology* **2018**, *13*, 882-889.
[22] A. Micsonai, É. Bulyáki, J. Kardos, in *Structural genomics: general applications*, Springer, **2020**, pp. 175-189.
